# *VIM-AS1*, which is regulated by CpG methylation, cooperates with IGF2BP1 to inhibit tumor aggressiveness via EPHA3 degradation in hepatocellular carcinoma

**DOI:** 10.1038/s12276-024-01352-6

**Published:** 2024-12-02

**Authors:** Su-hyang Han, Je Yeong Ko, Sungju Jung, Sumin Oh, Do Yeon Kim, Eunseo Kang, Myung Sup Kim, Kyung-Hee Chun, Kyung Hyun Yoo, Jong Hoon Park

**Affiliations:** 1 Laboratory of Biomedical Genomics, Department of Biological Sciences, Sookmyung Women’s University, Seoul, 04310 Republic of Korea; 2 Molecular Medicine Laboratory, Department of Biological Sciences, Sookmyung Women’s University, Seoul, 04310 Republic of Korea; 3 Department of Biochemistry and Molecular Biology, Graduate School of Medical Science, Brain Korea 21 Project, Yonsei University College of Medicine, Seoul, Republic of Korea; 4 Research Institute of Women’s Health, Sookmyung Women’s University, Seoul, 04310 Republic of Korea

**Keywords:** Liver cancer, Prognostic markers

## Abstract

Early tumor recurrence in hepatocellular carcinoma (HCC) remains a challenging area, as the mechanisms involved are not fully understood. While microvascular invasion is linked to early recurrence, established biomarkers for diagnosis and prognostication are lacking. In this study, our objective was to identify DNA methylation sites that can predict the outcomes of liver cancer patients and elucidate the molecular mechanisms driving HCC aggressiveness. Using DNA methylome data from HCC patient samples from the CGRC and TCGA databases, we pinpointed hypermethylated CpG sites in HCC. Our analysis revealed that cg02746869 acts as a crucial regulatory site for *VIM-AS1* (vimentin antisense RNA1), a 1.8 kb long noncoding RNA. RNA sequencing of HCC cells with manipulated *VIM-AS1* expression revealed *EPHA3* as a pathogenic target of *VIM-AS1*, which performs an oncogenic function in HCC. Hypermethylation-induced suppression of *VIM-AS1* significantly impacted HCC cell dynamics, particularly impairing motility and invasiveness. Mechanistically, reduced *VIM-AS1* expression stabilized *EPHA3* mRNA by enhancing the binding of IGF2BP1 to *EPHA3* mRNA, leading to increased expression of *EPHA3* mRNA and the promotion of HCC progression. In vivo experiments further confirmed that the *VIM-AS1‒EPHA3* axis controlled tumor growth and the tumor microenvironment in HCC. These findings suggest that the downregulation of *VIM-AS1* due to hypermethylation at cg02746869 increased *EPHA3* mRNA expression via a m6A-dependent mechanism to increase HCC aggressiveness.

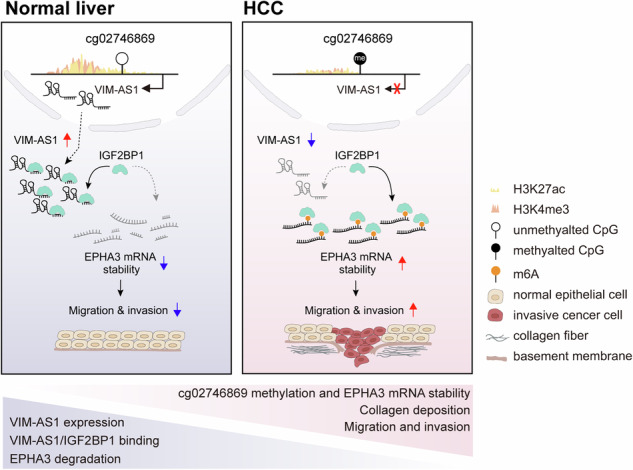

## Hypermethylation drives aggressiveness in liver cancer

Despite advancements in treatment, cancer remains a life-threatening disease that can recur (come back) and metastasize. Researchers found a knowledge gap in understanding how DNA methylation affects cancer progression. Researchers conducted an experiment to identify DNA methylation markers related to liver cancer prognosis. They used human liver cancer cell lines and analyzed DNA methylation and gene expression. The researchers discovered that hypermethylation of a specific DNA region in the *VIM-AS1* gene is linked to poor prognosis in liver cancer. They concluded that DNA methylation affects gene expression and cancer cell behavior. This finding could lead to new diagnostic and treatment strategies for liver cancer. Future research may explore how to target these epigenetic changes for better cancer therapies.

This summary was initially drafted using artificial intelligence, then revised and fact-checked by the author.

## Introduction

Despite advancements in treatment^1^, cancer remains a life-threatening disease^2^ that can recur and metastasize to various parts of the body^3^. Hepatocellular carcinoma (HCC) is characterized by high mortality and recurrence rates and is a significant cause of cancer-related death^4^. Tumor recurrence after liver resection plays a crucial role in determining patient survival, with early recurrence observed in approximately one-third of HCC patients within two years of surgery^5^. Microvascular invasion (MVI) is a critical risk factor for HCC recurrence, influencing tumor staging, prognosis, and treatment strategies because of its association with distant metastasis^6^. Moreover, aberrant expression of MVI-related markers such as PD-L1 and VEGF-A is linked to HCC malignancy, suggesting new therapeutic avenues^7^. These findings highlight MVI as a significant prognostic indicator for increased disease recurrence and reduced survival in patients with HCC.

Alterations in the tumor microenvironment influence the expression of key genes^8,9^, a process closely connected to changes in epigenetic regulatory elements such as DNA methylation and noncoding RNAs^10^. These findings highlight the importance of identifying epigenetic markers for early detection and prognosis assessment in HCC. Owing to its stability and specificity, DNA methylation shows promise as a diagnostic tool for early detection and prognosis evaluation in HCC^11^. Hypermethylation of the cg22538054 locus in the USP44 gene has been identified as a specific and sensitive biomarker for early-stage HCC^12^. Additionally, Luo and colleagues identified a set of DNA methylation signatures within HCC patient cohorts that outperform traditional prognostic biomarkers, including tumor stage and AFP levels, in predicting patient survival outcomes^13^.

Long noncoding RNAs (lncRNAs), key epigenetic regulators, play crucial roles in mediating environmental responses, influencing mRNA processing, and modulating stability, offering significant insights into pathological conditions^14^. Significant changes in lncRNA expression profiles have been documented in HCC, highlighting their critical functional roles in disease pathogenesis^15–17^. Notably, the tumor-suppressing lncRNA MEG3 inhibits cancer cell metastasis and angiogenesis by sponging *miR-145-5p*^18^. Furthermore, the lncRNA *ID2-AS1* controls *ID2* gene expression and reduces the invasion and spread of HCC cells by interacting with HDAC8^19^.

Currently, extensive studies are underway to comprehensively investigate epigenetic factors to enhance our understanding of cancer progression. In colorectal cancer, the upregulation of *GATA2-AS1* has been correlated with DNA hypermethylation within its gene body^20^, whereas the downregulation of *ZNF667-AS1* and MAFA-AS1 has been associated with changes in DNA methylation^21^. Similarly, in glioblastoma, the expression of *SNHG12*^22^ and *LINC02587*^23^ is influenced by CpG island methylation. However, in HCC, uncovering the complex regulatory relationship between DNA methylation and lncRNAs remains a crucial area of research. For example, CpG hypermethylation in the *MEG3* promoter region leads to *MEG3* transcriptional downregulation in HCC cells^24^.

In this study, our objective was to identify DNA methylation markers related to the prognostic outcomes of HCC and to examine the regulation of lncRNAs through methylation. Our findings suggest that epigenetic markers could be utilized for the diagnosis and treatment of HCC, as they have the potential to impact the aggressiveness of HCC cells.

## Materials and methods

### Cell culture

The human HCC cell lines HUH7, SNU449 and HEP3B were purchased from the Korean Cell Line Bank, and the HUH1 cell line was a gift from YJ Kim, Yonsei University, Korea. HUH7 and SNU449 cells were cultured in RPMI 1640 (LM011-03, Welgene) supplemented with 10% FBS. HEP3B cells were cultured in DMEM (LM001‒05, Welgene) supplemented with 10% FBS. HUH1 cells were cultured in DMEM (SH30243.01, HyClone) supplemented with 10% FBS. Human liver epithelial cells (THLE2; CRL-2706^TM^) were purchased from American Type Culture Collection (ATCC). THLE2 cells were cultured in bronchial epithelial cell growth medium (CC-3170, Lonza) supplemented with 10% FBS, 5 ng/mL epidermal growth factor and 70 ng/mL phosphoethanolamine.

### RNA preparation and reverse transcription

RNA was isolated using a Nucleospin RNA/Protein Kit (740933; Macherey-Nagel GmbH & Co.) following the manufacturer’s protocol. A mixture of RNA, oligo dT from Bioneer, dNTPs (U1205, U1215, U1225, and U1235, Promega), an RNase inhibitor (N211A, Promega), M-MLV 5X Reaction buffer (M531A, Promega), and M-MLV reverse transcript (M170A, Promega) was incubated at 42 °C for 1 h. cDNA synthesis was stopped at 70 °C. Quantitative real-time polymerase chain reaction (qRT‒PCR) was performed using the qPCRBIO SyGreen Blue Mix Lo-ROX (PB20.15-20, PCR Biosystems). The qRT‒PCR data were analyzed using comparative cycle threshold (2^-ΔΔCt^).

### Protein extraction and immunoblotting

Proteins were extracted from cells using a Nucleospin RNA/Protein Kit (740933, Macherey-Nagel GmbH & Co.) and separated on an 8–10% sodium dodecyl sulfate polyacrylamide electrophoresis gel. Proteins were then transferred onto a polyvinylidene fluoride membrane (W7031-270, GenDEPOT) and blocked with 5% skim milk (232100, BD) in PBST (0.1% Tween 20 in phosphate-buffered saline). The membrane was incubated overnight at 4 °C with an anti-EPHA3 antibody (sc-514209; Santa Cruz Biotechnology), an anti-β-actin antibody (ab8227; Abcam) and an anti-α-tubulin antibody (#3873; Cell Signaling Technology). After being washed with PBST, the membranes were incubated with horseradish peroxidase-conjugated secondary antibodies (ADI-SAB-100-J and ADI-SAB-300-J; Enzo) for 1 h at room temperature. The membrane was exposed to an enhanced chemiluminescence (ECL) substrate (34095, Thermo Scientific) and detected using an Amersham Imager 600 (GE Healthcare). Quantification was performed via ImageJ software (NIH).

### High-resolution melting (HRM)

Genomic DNA was extracted using a Quick-DNA Miniprep Plus Kit (D4068, Zymo Research) according to the manufacturer’s instructions. An EZ DNA Methylation-Gold Kit (D5006, Zymo Research) was used for bisulfite treatment of the genomic DNA according to the manufacturer’s protocol. Bisulfite-treated DNA was amplified using polymerase chain reaction (PCR) with HotStar Taq Plus DNA polymerase (203203; Qiagen). The primers were designed using MethPrimer 2.0. Methylation estimates for the target CpG sites were included in the amplified region. The PCR products were subjected to high-resolution melting analysis. Following PCR, the melting curves were analyzed using ImageJ 1.53k (NIH).

### Dual-luciferase reporter assay

The human *EPHA3* untranslated region (3′ UTR), including the predicted N6-methyladenosine (m6A) site and its mutated sequence, was inserted into the pmirGLO plasmid. This luciferase reporter construct was transfected into HUH1 cells using the FuGene (E2311, Promega) transfection reagent. After transfection, luciferase activity was measured using a dual-luciferase reporter assay system (E1910, Promega) according to the manufacturer’s instructions. In addition, the human *EPHA3* 3′UTR, including the predicted m6A site and its mutated sequence, was inserted into the pmirGLO plasmid (E1330, Promega) via an In-Fusion HD cloning kit (#639649, TAKARA). The *EPHA3* 3’UTR sequence was generated using PCR of RNA from HEK293T cells. The primers used to amplify the *EPHA3* 3’UTR wild-type sequence were as follows: 5’-TAGCCTCGAGTCTAGATCAGCGTAAAATGGTGAAGAACT-3’ (forward) and 5’-GCAGGTCGACTCTAGATAGTAAATATGGCCAGATAGTTTGA-3’ (reverse) with an XbaI site. Primers containing the mutation site were used to generate the 3′ UTR mutated sequence.

### Construction of dCas9 expression plasmids

pdCas9-DNMT3A-EGFP was a gift from Vlatka Zoldoš (Addgene, Plasmid # 71666). The guide RNA (gRNA) sequences were annealed and inserted into the plasmid via the BbsI sites and T4 DNA ligase. pPlatTET-gRNA2 was a gift from Izuho Hatada (Addgene, Plasmid #82559). Two 60-mer oligonucleotides containing the gRNA sequence were annealed using Phusion DNA polymerase to generate a 100 bp dsDNA fragment. The plasmid was linearized with AflII, and the gRNA fragment was incorporated via Gibson assembly. The small guide RNA (sgRNA) sequence, its target cg site, and the primer set used for gRNA synthesis are listed in Supplementary Fig. 1b. THLE2 and HUH1 cells were transfected with Lipofectamine Lipo3000 (L3000015, Life Technologies) according to the manufacturer’s instructions, and 1 d after transfection, the cells were passaged to plates for the assay.

### Construction of human *VIM-AS1* and *EPHA3* expression plasmids

Human *VIM-AS1* cDNA was generated using PCR. The primers used were 5′-CTGCAGCTAGCTCGAGTTCTCCCGGAGGCGCATG-3′ (forward) and 5′-CGGTAGAATTCTCGAGTTTTTTTTTAGCATATCCAAAAATG-3′ (reverse) with a NotI site. After the plasmid was cleaved with NotI, the PCR product containing full-length *VIM-AS1* cDNA was inserted into the pHAGE-puro plasmid vector (Addgene plasmid #118692). HUH1 cells were stably transduced with a lentivirus carrying *VIM-AS1*. RT‒PCR was used to quantify *VIM-AS1* expression. Human *EPHA3* cDNA was generated via PCR. The primers used were 5′-CGGGCTGCAGGAATTCATGGATTGTCAGCTCTCCATCC-3′ (forward) and 5′-GCT TGATATCGAATTCTTACACGGGAACTGGGCC-3′ (reverse) with a NotI site. After the plasmid was cleaved with NotI, the PCR product containing full-length *EPHA3* cDNA was inserted into the pLECE-GFP-Luc plasmid vector. HUH1_empty vector control cells and HUH1_*VIM-AS1*-overexpressing cells were stably transduced with lentivirus carrying *EPHA3*.

### *VIM-AS1* knockdown using siRNA

The cells were seeded into 12-well plates in growth medium until 60–80% confluency was achieved, and a mixture containing siRNAs against *VIM-AS1* (siRNA ID: n542380, Thermo Fisher Scientific), RNAiMAX (13778150, Thermo Fisher Scientific) reagent, and Opti-MEM (31985-070, Gibco) medium was added to the cell medium. The cells were harvested for qPCR analysis to determine the efficiency of *VIM-AS1* inhibition.

### RNA immunoprecipitation (RIP) and m6A RNA immunoprecipitation

The cell lysates were immunoprecipitated using magnetic beads (10002D and 10004D, Thermo Fisher Scientific) precoated with an anti-IGF2BP1 antibody, anti-m6A antibody (68055-1-Ig, Proteintech), or anti-mouse IgG (CS200581, Sigma‒Aldrich). After treatment with proteinase K (25530-049, Invitrogen), the RNA was eluted and purified for qPCR. The enrichment data were normalized to the following input: % input = 1/10 × 2Ct[IP] - Ct[input].

### Actinomycin D assay

The cells were seeded in 6-well plates before treatment with actinomycin D (A9415, Sigma‒Aldrich) or dimethylsulfoxide. HUH1_empty vector control cells and HUH1_*VIM-AS1*-overexpressing cells were treated with 10 μg/mL actinomycin D for 4 and 24 h. RNA was extracted, and its stability was tested via quantitative reverse transcription‒PCR (qRT‒PCR).

### Fluorescence in situ hybridization (FISH) and immunofluorescence (IF)

The cells were fixed with 3.7% formaldehyde, permeabilized with 70% EtOH, and washed with wash buffer A (SMF-WA1-60; BioResearch Technology). The samples were subsequently incubated overnight with a Cy3-labeled *VIM-AS1* probe mixture (SMF-1025-5, Bioresearch Technologies) or a Cy5-labeled *EPHA3* probe mixture (SMF-1065-5, Bioresearch Technologies) with or without the IGF2BP1 antibody (22803-1-AP, Proteintech) at 37 °C. The cells were incubated with an Alexa Fluor^TM^ 647-conjugated secondary antibody (A-21245, Invitrogen) and DAPI (D9564, Sigma‒Aldrich) for 1 h at 37 °C. The cells were subsequently washed with wash buffer B (SMF-WB1-20; BioResearch Technology) and mounted with a mounting solution. Images were captured via a confocal laser microscope (Carl Zeiss). To measure the fluorescence intensity of the cells, the cells in the image were compartmentalized, and the fluorescence intensity was measured using ZEN blue 3.4.

### Hematoxylin and eosin, Sirius Red, Masson’s trichrome and immunofluorescence staining

Tumor tissues were fixed in 10% formalin (F0196RD; BYLABS) and paraffin-embedded. The tissue sections were stained with hematoxylin and eosin (H&E), Sirius red, and Masson’s trichrome for morphological analysis. Immunofluorescence was conducted on formalin-fixed, paraffin-embedded tissue sections. The sections were deparaffinized and rehydrated via Histoclear (HS-202, Ted Pella Inc.). Antigen retrieval was performed using a Borg Decloaker (BRR1000AG1; Biocare Medical), and the samples were boiled for 15 min. Nonspecific binding was blocked with goat serum (S-1000, Cole-Parmer) for 10 min at room temperature. The sections were incubated with anti-α-SMA (#19245, Cell Signaling) or anti-CD31 (#3528, Cell Signaling) antibodies overnight, followed by incubation with 488 secondary antibodies (#A-11029 and #A-11034, Invitrogen) and DAPI staining solution. Images were analyzed using Axio Vert A1 (Carl Zeiss) and LSM700 (Carl Zeiss) at the Chronic and Metabolic Diseases Research Center, Sookmyung Women’s University.

### Bisulfite sequencing

Bisulfite-treated DNA was amplified using TA cloning. The PCR products were subsequently inserted into the pCR^TM^4-TOPO vector (450030, Thermo Fisher Scientific), and 10 clones were sequenced by Bionics (Seoul, Korea). The percentage of methylation was calculated as the number of methylated cytosines divided by the total number of cytosines [methylated cytosines and thymines (unmethylated cytosines)].

### Methylation profiling

We performed a reanalysis of DNA methylome data from HCC patient samples obtained from databases (CGRC and TCGA) to identify CpG sites that are hypermethylated in HCC. The CGRC methylation data were obtained from patients with HCC (125 matched normal and 180 tumor tissues). TCGA methylation data were obtained from the TCGA-LIHC cohort (50 nontumor and 379 tumor tissues)^25^. Methylation analyses were performed as previously described^26^.

### ChIP-seq analysis

After sequencing on the Illumina platform, raw sequence files (.fastq) were used to perform quality control checks via *FastQC* (http://www.bioinformatics.babraham.ac.uk/projects/fastqc). The adapter sequence was then removed, and the low-read, low-quality sequences were trimmed using *Cutadapt*^27^. Trimmed clean read sequence files were mapped to the human reference genome (version hg19) using *Bowtie2*^28^. To profile the mapping results (*.bam*), we created a tag directory, UCSC files, peak calling, annotated peaks, and motif search using *HOMER* (*makeTagDirectory*, *makeUCSCfile*, *findPeaks*, annotatePeaks, and *findMotifs*, respectively)^29^. The ChIP-seq profiling data were visualized using the Integrative Genomics Viewer (IGV)^30^.

### RNA-seq analysis

After the raw sequence data were generated on the Illumina platform, the processes of quality control and trimming were similar to those used for ChIP-seq analysis. Gene expression levels were calculated from the trimmed clean read sequence files by mapping them to the human reference genome (version hg19) using *RSEM*^31^ with a *STAR aligner*^32^. Then, via the *Bioconductor package DESeq2*^33^ in *R*, the count table was normalized, and the gene expression level for each group was compared to identify differentially expressed genes (DEGs). Gene Ontology (GO) analysis was performed via gene set enrichment analysis (GSEA)^34^ to identify the biological functions of the DEGs using a normalized count table.

### In vivo tumorigenesis assay

BALB/c nu/nu mice were subcutaneously injected with 8 × 10^6^ tumor cells (control, *VIM-AS1*-overexpressing or *VIM-AS1*- and *EPHA3*-overexpressing cells). Tumor sizes were measured on days 29, 34, and 40, and tumor volumes were calculated using the following formula: (length × width^2^)/2. The mice were euthanized 6 weeks after experimental initiation. All animals were handled in accordance with the institutional guidelines and approved by the Yonsei University Institutional Animal Care and Use Committee (IACUC 2020-0090).

### Survival analyses

The whole-genome DNA methylation profiles of 369 primary tumor samples were obtained from TCGA with clinical survival information^25^. The correlation between beta values and overall survival of patients with HCC was analyzed using the “survival” package in R. The correlation between gene expression levels and the survival of patients with HCC was analyzed via the online Kaplan–Meier plotter^35^ and R.

### Statistical analyses

The results of the statistical analyses are presented as mean and standard deviation. Student’s t test was performed using GraphPad Prism 5. Statistical significance was set at p < 0.05.

## Results

### Methylation status of cg02746869 regulated *VIM-AS1* expression in HCC

**Fig. 1:**
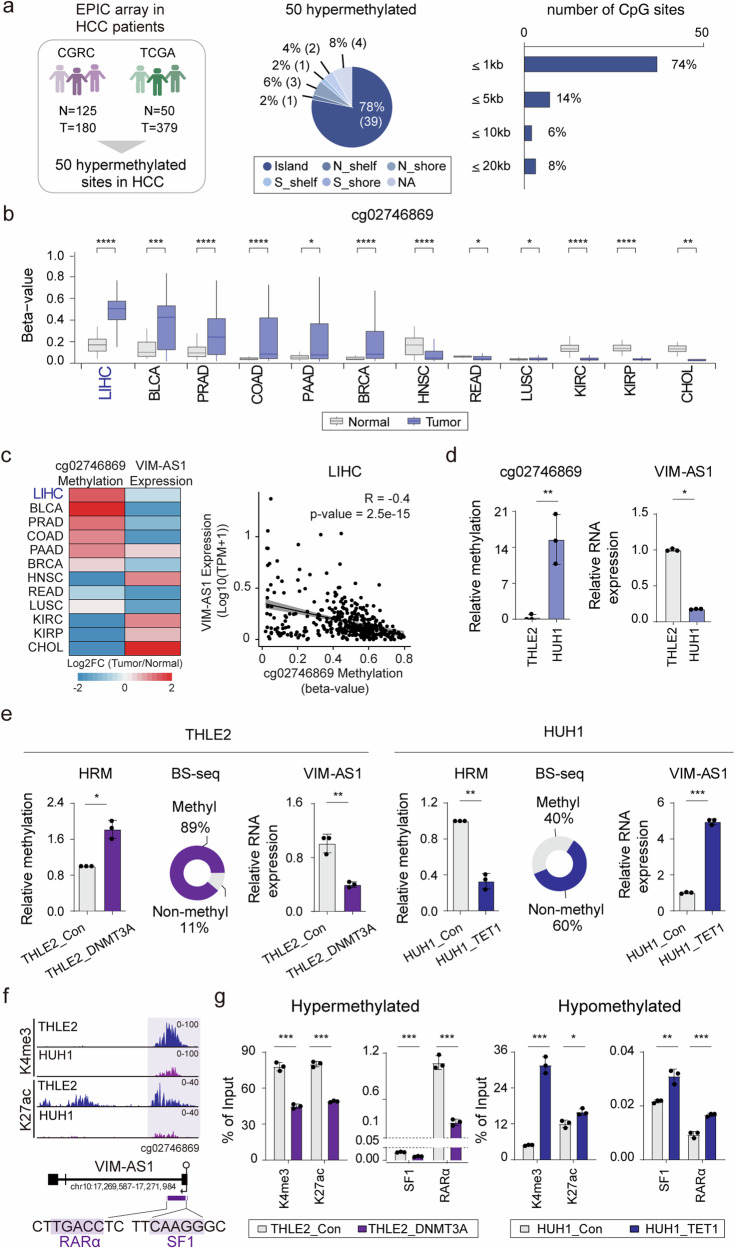
DNA methylation profiling reveals hypermethylated cg02746869 in *VIM-AS1* of HCC patients. **a** Schematic illustration showing the identification of 50 hypermethylated sites in HCC on the basis of methylome data derived from the CGRC and TCGA (left). Fifty hypermethylated sites were located on CpG islands, shores, shelves, and the open sea (middle). Distribution of the distance between the 50 hypermethylated sites and the TSS (right). **b** Analysis of the beta values for cg02746869 in multiple malignancies. **c** Heatmap representing the pattern of *VIM-AS1* expression and cg02746869 methylation in various types of cancer (left). Correlation analysis of cg02746869 beta values and VIM-AS1 expression levels (right). **d** Methylation of cg02746869 and associated *VIM-AS1* expression in THLE2 and HUH1. **e** Increased methylation levels were confirmed using HRM and BS-seq, and decreased *VIM-AS1* expression was detected in the DNMT3A experimental set. A decrease in methylation was confirmed using HRM and BS-seq, and *VIM-AS1* expression was verified in the TET1 experimental set. **f** Genomic viewer showing enrichment of active markers at cg02746869 in THLE2 and HUH1. **g** ChIP‒qPCR for assessing histone activity and TF binding following the methylation and demethylation of cg02746869. Con = empty vector, DNMT3A = hypermethylated cg02746869, TET1 = hypomethylated cg02746869. *p < 0.05, **p < 0.01, ***p < 0.001, ****p < 0.0001.

To investigate differentially methylated regions in liver cancer tissues, we performed DNA methylation profiling across the CGRC (125 adjacent nontumor tissues and 180 tumor tissues) and TCGA (379 tumor tissues and 50 adjacent nontumor tissues) cohorts. Among the top 50 sites that were hypermethylated in liver cancer relative to controls, 78% (39) were present in CpG islands (Fig. 1a). We further analyzed the locations of the hypermethylated sites on the basis of their distance from the transcription start site (TSS) (Fig. 1a). Approximately 70% (37) of the hypermethylated sites were located within 1 kb, suggesting that the DNA methylation of CpGs within promoters affects gene silencing mechanisms, resulting in gene downregulation^13^. Compared with other cancers, cg02746869 presented the highest level of hypermethylation in liver cancer (Fig. 1b). Next, to determine whether methylation status contributes to gene expression, we analyzed the expression of *VIM-AS1* (vimentin antisense RNA 1), a 1.8 kb long noncoding RNA associated with cg02746869 in the TCGA dataset. In cancers with high beta values for cg02746869, such as LIHC, *VIM-AS1* expression was downregulated, whereas in tumor types with low beta values, such as CHOL, *VIM-AS1* expression was upregulated (Fig. 1c). Furthermore, our correlation analysis of the TCGA-LIHC cohort revealed that increased methylation of cg02746869 corresponded with decreased expression of *VIM-AS1* (Fig. 1c). In addition, higher methylation of cg02746869 tended to be associated with a poorer prognosis, and reduced *VIM-AS1* expression was significantly associated with a decreased survival rate of patients with HCC (Supplementary Fig. 1a). These data suggested that hypermethylation at the *VIM-AS1* locus and its subsequent reduced expression were associated with a poor prognosis of HCC patients.

In agreement with the clinical data, hypermethylation of cg02746869 was observed in HUH1 human liver cancer cell line, compared with normal human liver cells, THLE2, with a corresponding decrease in *VIM-AS1* expression in HUH1 cells (Fig. 1d). We aimed to change the methylation of the sites within *VIM-AS1* and determine whether *VIM-AS1* expression could be modulated. We used the CRISPR-dCas9-DNMT3A system to alter the methylation status of cg02746869 in THLE2, which decreased *VIM-AS1* levels (Supplementary Fig. 1b and Fig. 1e). Conversely, we used the CRISPR-dCas9-TET1 system to induce demethylation of cg02746869 in HUH1, HUH7, SNU449 and HEP3B. Interestingly, hypomethylation of cg02746869 increased the expression of *VIM-AS1* and two genes with which it overlaps, but it had no effect on *VIM* (vimentin) mRNA expression (Supplementary Fig. 1b–d and Fig. 1e). These findings demonstrated that altered DNA methylation functions as an upstream regulator of *VIM-AS1*.

To determine whether the status of CpG methylation alters histone modifications, we performed ChIP-seq for H3K27ac and H3K4me3, which are active histone markers. We observed a significant decrease in the enrichment of active histone markers in HUH1 cells with hypermethylated DNA compared with that in THLE2 cells (Supplementary Fig. 1e and Fig. 1f). Furthermore, we investigated the potential binding of SF1 and RARα to hypermethylated regions in HCC (Supplementary Fig. 1f). To determine whether histone activities and the binding of transcription factors (TFs) are regulated by CpG methylation, we performed ChIP‒qPCR after targeted methylation modulation using dCas9-DNMT3A and TET1. The results showed that cg02746869 methylation via DNMT3A in THLE2 decreased histone activity and the binding of SF1 and RARα. cg02746869 demethylation via TET1 increased histone activity and the binding of TFs to HUH1 (Fig. 1g). These results indicated that the modulation of the DNA methylation status at *VIM-AS1* regulated histone activity and the binding of TFs.

### Overexpressing *VIM-AS1* inhibited the aggressiveness of cancer cells

**Fig. 2:**
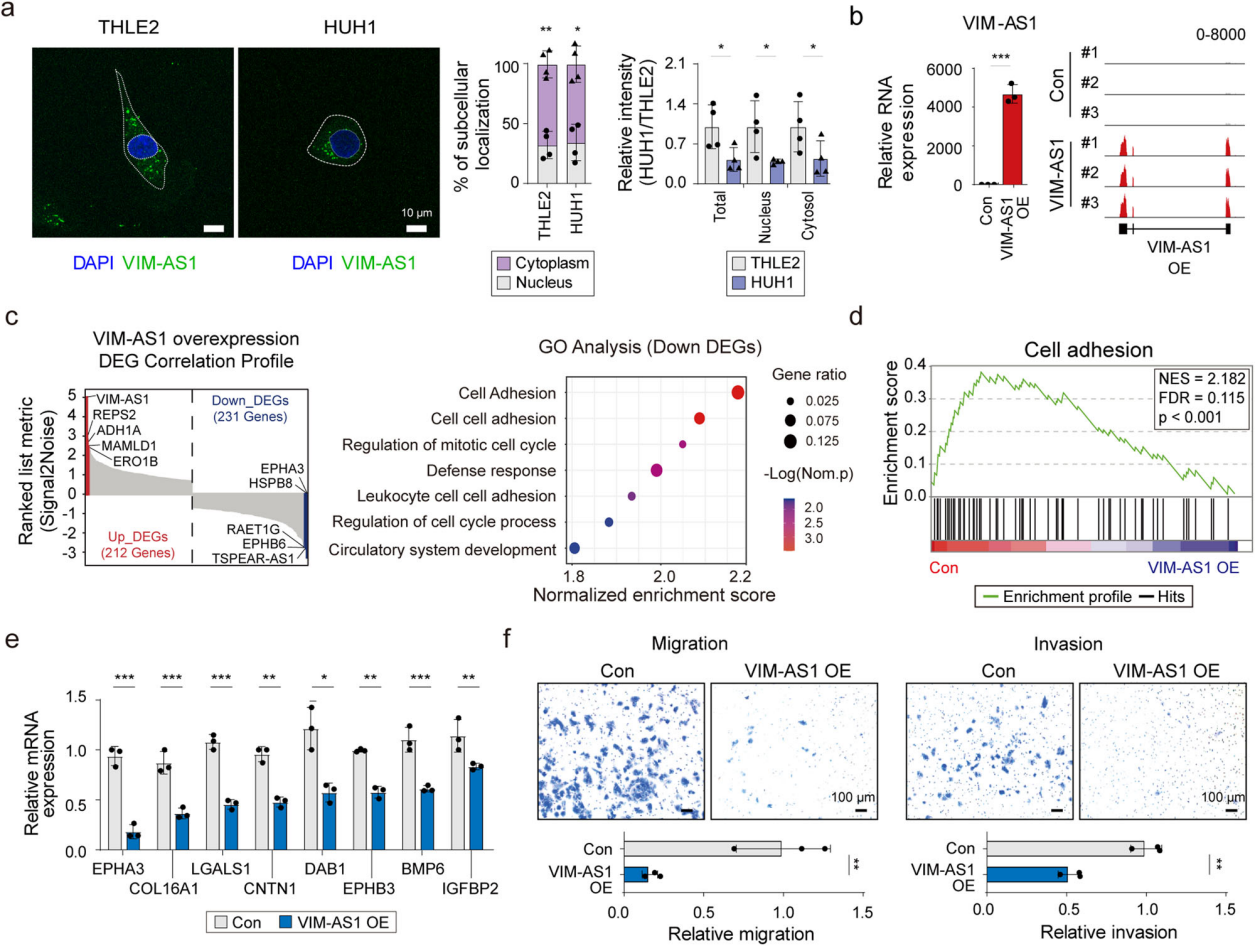
Increased levels of *VIM-AS1* globally downregulate genes related to cell adhesion. **a** Representative image of *VIM-AS1* localization was detected using FISH (left). Relative intensity of *VIM-AS1*, indicating the percentage of subcellular localization (middle). Comparison of the intensity of each subcellular localization between THLE2 and HUH1 (right). **b** Validation of *VIM-AS1*-overexpressing cells via qRT‒PCR (left) and RNA-seq (right). **c** DEG correlation profile of up- and downregulated genes after overexpressing *VIM-AS1* (left). Red = top 5 upregulated genes; blue = top 5 downregulated genes. GO analysis of DEGs downregulated by *VIM-AS1* (right). **d** GSEA results of downregulated DEGs associated with cell adhesion. **e** qRT‒PCR of cell adhesion-associated genes in *VIM-AS1*-overexpressing cells. **f** Representative images and quantification of migration and invasion assays. Con = Control, *VIM-AS1* OE = *VIM-AS1* overexpression, *p < 0.05, **p < 0.01, ***p < 0.001.

We analyzed the localization of *VIM-AS1* to elucidate its cellular function. Fluorescence in situ hybridization (FISH) revealed that *VIM-AS1* was located mainly in the cytoplasm of HCC and normal liver cells (Fig. 2a). Consistent with the results of the transcriptome and qPCR analyses, the relative intensity of *VIM-AS1* was lower in HUH1 cells than in THLE2 cells at each location (Fig. 2a and Supplementary Fig. 2a). These results suggest that *VIM-AS1*, which is regulated by methylation and is present in the cytoplasm, is possibly involved in posttranscriptional gene regulation in HCC.

To determine the role of *VIM-AS1* in HCC progression, RNA-seq was performed using *VIM-AS1*-overexpressing HUH1 cells (Fig. 2b). In total, 443 significantly differentially expressed genes (DEGs) were identified, 212 of which were upregulated and 231 of which were downregulated upon *VIM-AS1* elevation (Fig. 2c and Supplementary Table 1). Considering that *VIM-AS1* expression is reduced in HCC, we focused on the functions of genes downregulated upon *VIM-AS1* upregulation to understand their inhibitory effects on cancer progression. Gene Ontology (GO) analysis indicated that genes downregulated with increasing *VIM-AS1* expression were involved in cell adhesion (Fig. 2c and Supplementary Fig. 2b). This association was consistently observed via GO analysis using KEGG and BioPlanet (Supplementary Fig. 2c). In addition, gene set enrichment analysis (GSEA) revealed that genes related to cell adhesion were downregulated with increasing *VIM-AS1* expression (Fig. 2d). Using qRT‒PCR, we confirmed that the mRNA expression of genes involved in cell adhesion, including *EPHA3, COL16A1, LGALS1*, and *CNTN1*, was significantly downregulated in cells overexpressing *VIM-AS1* (Fig. 2e).

Next, we performed migration and invasion assays to determine whether the overexpression of *VIM-AS1*, which regulates genes involved in cell adhesion, could modulate the aggressiveness of HCC cells (Fig. 2f). The migratory and invasive abilities of HCC cells decreased with increasing *VIM-AS1* expression. Conversely, reducing *VIM-AS1* in THLE2 cells increased cell migration and invasion (Supplementary Fig. 2d). Interestingly, methylation of cg02746869 increased the migratory and invasive abilities of THLE2 cells (Supplementary Fig. 2e), while its demethylation attenuated these abilities in HUH1 cells (Supplementary Fig. 2f). These results indicated that *VIM-AS1* regulated the migration and invasion of HCC cells by modulating the expression of genes associated with cell adhesion.

### *VIM-AS1* regulated the stability of *EPHA3* mRNA

**Fig. 3:**
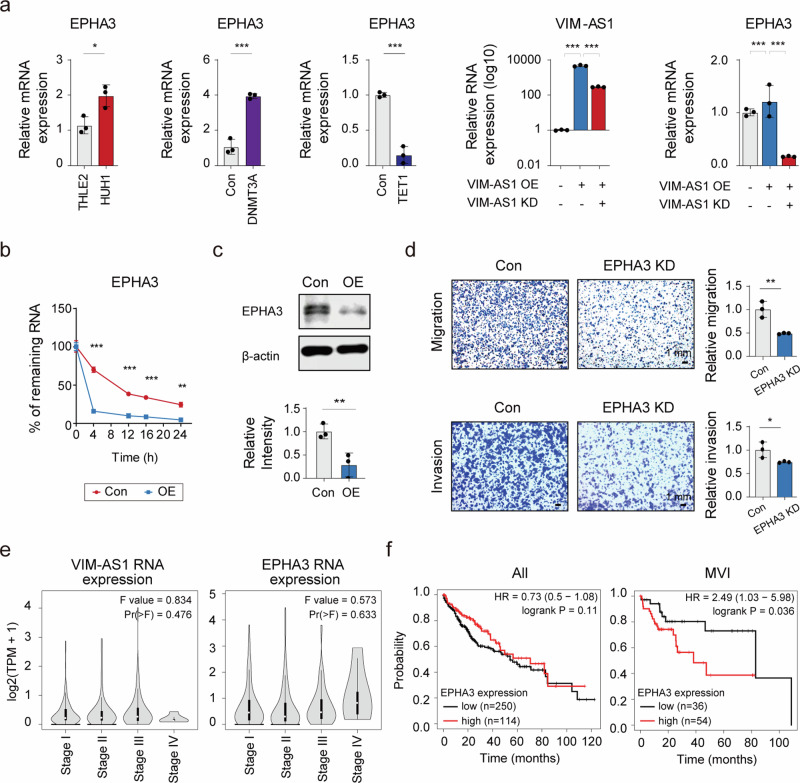
*VIM-AS1* regulates the stability of *EPHA3* mRNA, which is associated with poor prognosis in HCC. **a** Relative mRNA expression of *EPHA3* in THLE2, HUH1 and samples with modified methylation. Changes in *EPHA3* mRNA expression are shown in response to the modulation of *VIM-AS1*. *VIM-AS1* OE = *VIM-AS1* overexpression, *VIM-AS1* KD = *VIM-AS1* knockdown. **b** RNA stability assay for *EPHA3* mRNA after actinomycin D treatment of HUH1 cells. Con = Control, OE = *VIM-AS1* overexpression. **c** Immunoblot of EPHA3 protein after *VIM-AS1* overexpression. **d** Cell migration and invasion assays following *EPHA3* mRNA knockdown. *p < 0.05, **p < 0.01, ***p < 0.001. Con = Control, *EPHA3* KD = *EPHA3* knockdown. **e** *VIM-AS1* and *EPHA3* RNA expression according to the stage was analyzed using GEPIA. **f** Probability of survival according to *EPHA3* mRNA in all patients with HCC and with MVI analyzed using Kaplan–Meier plotter.

To further elucidate the regulatory mechanism of *VIM-AS1*, we focused on *EPHA3* mRNA among the genes related to cell adhesion, the expression of which was most significantly reduced upon *VIM-AS1* overexpression (Fig. 2e). *EPHA3* mRNA was upregulated in HUH1 compared with THLE2, and its expression was correlated with the methylation status of cg02746869 (Fig. 3a). Furthermore, *EPHA3* mRNA expression was inversely related to *VIM-AS1* expression (Fig. 3a). These results, along with the observation that inhibition of *VIM-AS1* expression in THLE2 cells restored the mRNA expression of *EPHA3* (Supplementary Fig. 3a), indicated that *VIM-AS1* directly regulated the expression of *EPHA3* mRNA.

On the basis of the involvement of pathogenic lncRNAs in regulating mRNA stability in the cytoplasm^36^, we examined whether *VIM-AS1* regulated the stability of *EPHA3* mRNA (Fig. 3b). Control and *VIM-AS1*-overexpressing cells were treated with actinomycin D, and *EPHA3* mRNA expression was examined. We observed that *VIM-AS1* overexpression increased the degradation rate of *EPHA3* mRNA at all time points (Fig. 3b), which in turn reduced the EPHA3 protein level (Fig. 3c). These data suggest that *VIM-AS1* affects *EPHA3* mRNA expression by regulating the stability of its mRNA in HCC. As *EPHA3* mRNA is a cell adhesion-related gene, we validated the biological function of EPHA3 in HCC. Cell migration and invasion were significantly inhibited by a reduction in *EPHA3* mRNA expression (Fig. 3d and Supplementary Fig. 3b), which was consistent with the phenotype induced by the upregulation of *VIM-AS1* expression in HCC cells.

Furthermore, analysis of HCC patient data from TCGA revealed that *VIM-AS1* expression decreased, whereas *EPHA3* mRNA expression increased with disease progression (Fig. 3e). Survival analysis did not reveal any significant correlation of *EPHA3* mRNA with the overall survival of patients with HCC; however, in patients with MVI, higher *EPHA3* mRNA was linked to shorter survival (Fig. 3f). These results suggest that *VIM-AS1*-regulated *EPHA3* mRNA expression is correlated with a poor HCC prognosis.

### *EPHA3* mRNA stability was determined by the binding of IGF2BP1 to the m6A site

**Fig. 4:**
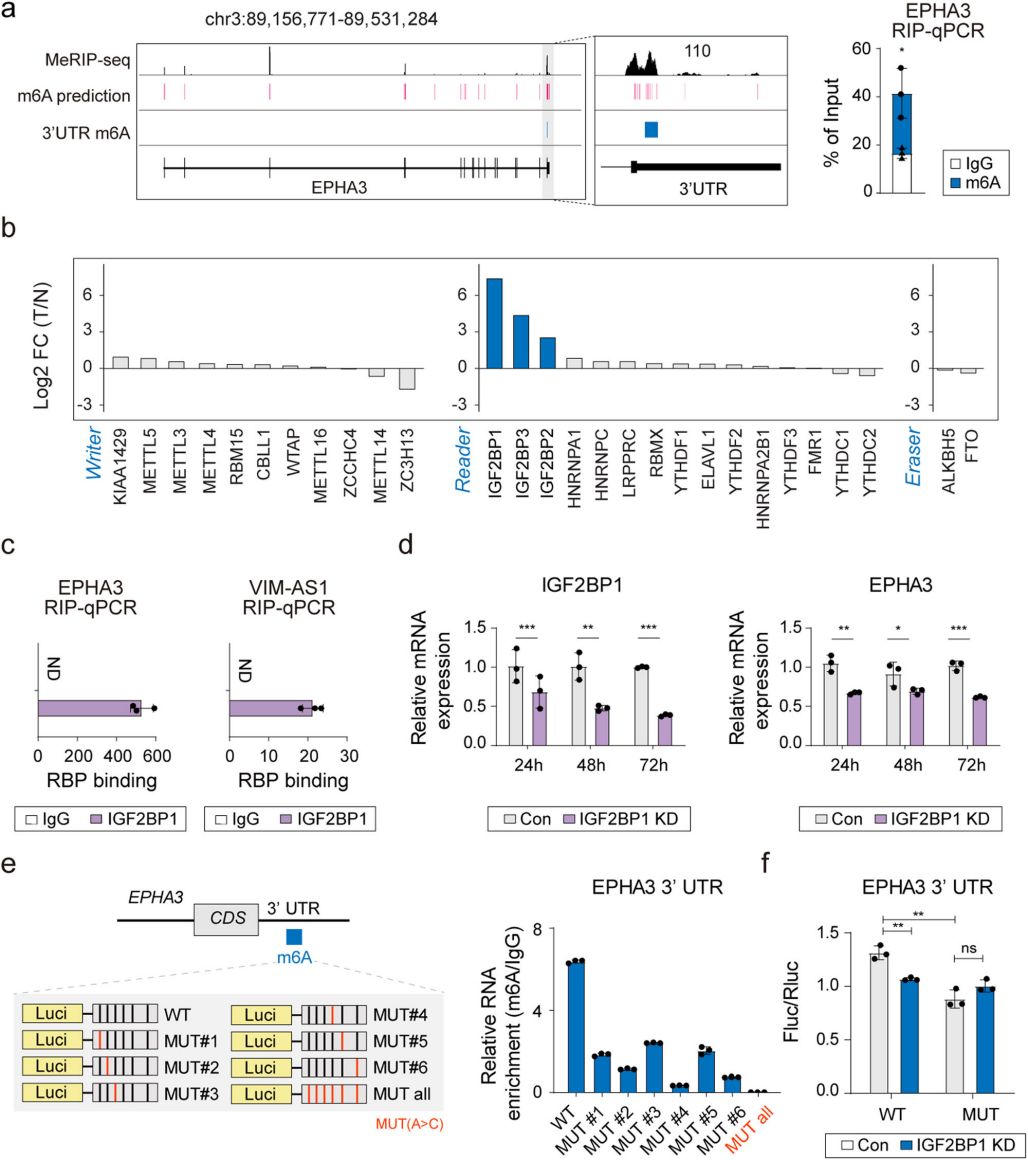
IGF2BP1 impairs the stability of *EPHA3* mRNA in a m6A-dependent manner. **a** Genome browser snapshot showing the m6A modification sites of *EPHA3* MeRIP-seq in HEK293T cells from GSE928399 (top), prediction of m6A sites using RM2Target (middle) and experimental targeting regions of 3′UTR m6A sites (bottom). The results of MeRIP-qPCR for the 3′ UTR of *EPHA3*. **b** Differential expression of m6A modifiers in HCC tumor tissue (T) relative to normal tissue (N); FC = fold change. **c** RIP‒qPCR assays showing *EPHA3* and *VIM-AS1* RNA bound to IGF2BP1 in HUH1 cells. **d** Effect of IGF2BP1 silencing on *EPHA3* mRNA expression. **e** MeRIP‒qPCR analysis of *EPHA3* 3′ UTR wild-type (WT) and mutant (MUT) constructs, including the m6A sites. **f** Luciferase assays using WT and MUT constructs after IGF2BP1 silencing. ND = not detected, Con = control, *IGF2BP1* KD = *IGF2BP1* knockdown. *IGF2BP2* KD = *IGF2BP2* knockdown. WT = wild type, MUT = mutant, *p < 0.05, **p < 0.01, ***p < 0.001, ns = not significant.

To investigate the mechanism underlying the regulation of *EPHA3* mRNA stability in HCC, we aimed to identify the m6A modification site at the 3′ UTR that may act as a binding site for specific proteins, known as m6A regulators^37,38^. Using public methylated RNA immunoprecipitation (MeRIP)-seq data (GSM928399) in conjunction with RM2Target, we pinpointed potential sites of m6A modification within the 3′ UTR of *EPHA3* (Fig. 4a). The results of this prediction were further corroborated by the results of the MeRIP‒qPCR assay, which demonstrated the abundant presence of m6A modifications in HUH1 cells (Fig. 4a). As m6A modification is regulated by m6A-binding proteins, we evaluated the expression of m6A writers, readers, and erasers in samples from patients with HCC (GSE77314). Considering the elevated expression of IGF2BP1, 2, and 3 in HCC tissues relative to normal tissues (Fig. 4b), we posited that these factors modulated the stability of *EPHA3* mRNA, a hypothesis supported by predictive analyses, which indicated robust binding affinity of IGF2BPs to *EPHA3* mRNA (Supplementary Fig. 4a). Moreover, considering the regulatory influence of *VIM-AS1* on *EPHA3* mRNA, analysis of the *VIMAS1* protein interaction network revealed the ability of *VIM-AS1* to interact with IGF2BP1/2 (Supplementary Fig. 4a). We confirmed the binding of these RNA-binding proteins (RBPs) to both *EPHA3* mRNA and *VIM-AS1* via RIP-qPCR (Fig. 4c and Supplementary Fig. 4b).

Next, to identify the RBPs that regulate the mRNA expression of *EPHA3*, we used siRNAs to inhibit IGF2BP1/2 and quantified the changes in *EPHA3* mRNA levels using qRT-PCR. Interestingly, only IGF2BP1 knockdown significantly decreased *EPHA3* mRNA expression (Fig. 4d and Supplementary Fig. 4c). This finding was also confirmed in HUH7 cells, which presented a decrease in *EPHA3* mRNA levels upon a reduction in IGF2BP1 (Supplementary Fig. 4d). There are several pieces of evidence to support our findings. Although IGF2BP2 can bind to mRNAs, it does not necessarily regulate their expression^39^. Additionally, acetylation of IGF2BP2 can either increase or decrease the expression of target genes^40^. These findings align with our findings that while both IGF2BP1 and IGF2BP2 can bind to *EPHA3* mRNA, only IGF2BP1 is involved in reducing *EPHA3* mRNA expression. Notably, IGF2BP1 was found to be overexpressed in HCC (Supplementary Fig. 4e) and to bind to multiple sites on *VIM-AS1* (Supplementary Fig. 4f). Furthermore, our study confirmed that IGF2BP1 regulates the stability of *EPHA3* mRNA, whereas IGF2BP2 does not (Supplementary Fig. 4g). These results suggest that the binding of IGF2BP1 to the m6A modification site is crucial for regulating the stability of *EPHA3* mRNA.

Furthermore, we elucidated the contribution of m6A-mediated regulation by IGF2BP1 to the stability of *EPHA3* mRNA by using luciferase assays with constructs bearing wild-type and mutant *EPHA3* 3’ UTR m6A sites. Figure 4e shows that, compared with the other mutant constructs, all of the m6A mutants (MUTs) presented the greatest decrease in m6A modification. Therefore, we used this particular construct for the luciferase assay (Fig. 4f). The results revealed that the activity of the 3′ UTR m6A sites was downregulated when the sites were mutated. Moreover, silencing of IGF2BP1 reduced the luciferase activity of cells transfected with the wild-type *EPHA3* 3’ UTR m6A sites, whereas the activity in cells transfected with the mutant EPHA3 appeared to remain unchanged. These results suggest that the regulatory axis comprising m6A modification at the *EPHA3* 3′ UTR and IGF2BP1 controls *EPHA3* mRNA levels in HCC.

### *VIM-AS1* interferes with the binding of IGF2BP1 to *EPHA3*

**Fig. 5:**
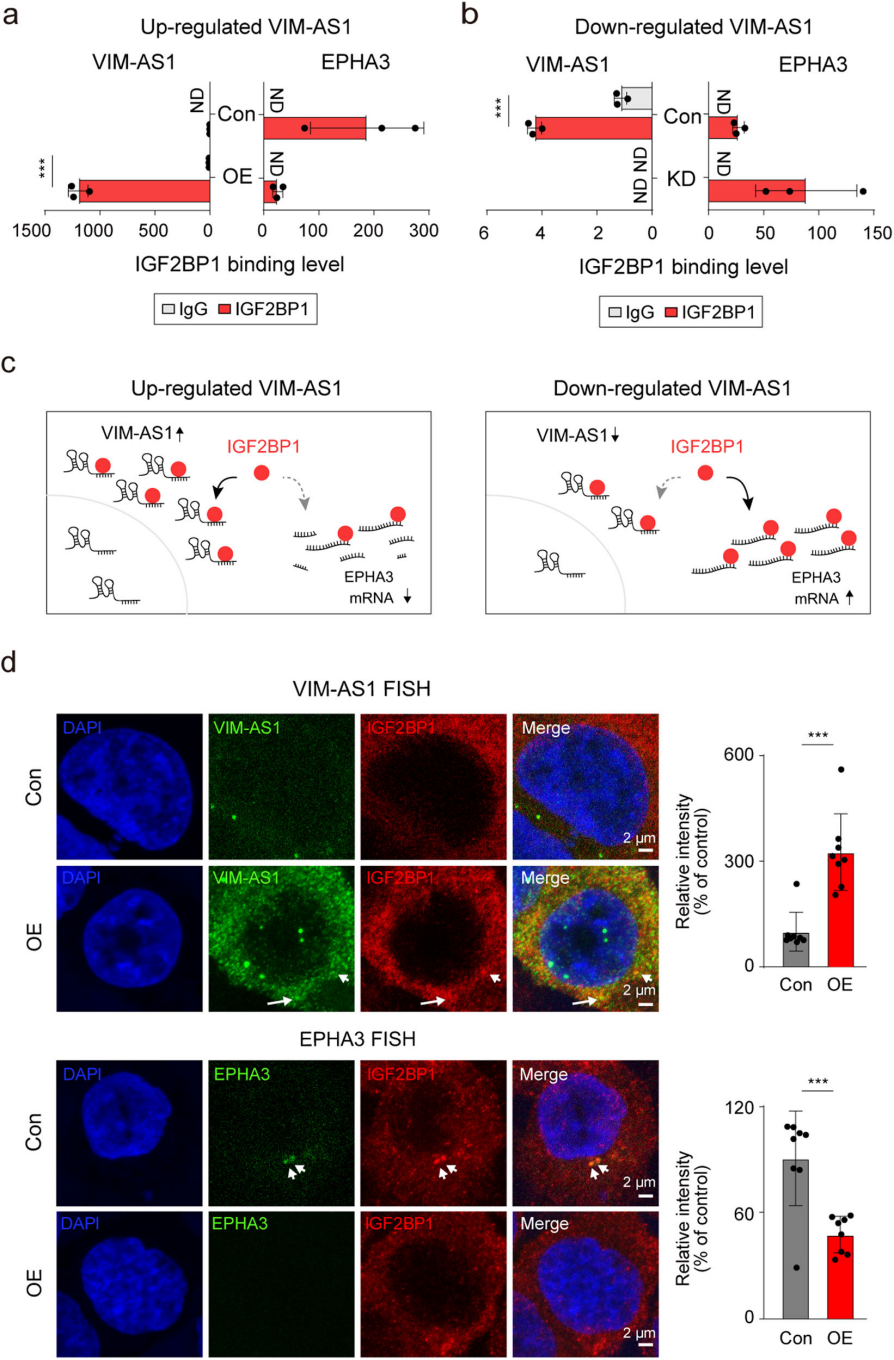
*VIM-AS1* regulates *EPHA3* mRNA expression by inhibiting the interaction between IGF2BP1 and *EPHA3* mRNA. RIP-qPCR assay showing the binding affinity of *VIM-AS1* and *EPHA3* mRNAs for IGF2BP1 following the overexpression of *VIM-AS1* in HUH1 cells (**a**) and *VIM-AS1* knockdown in THLE2 cells (**b**). **c** A schematic illustrating how the effect of increasing/decreasing *VIM-AS1* expression on *EPHA3* mRNA is mediated by IGF2BP1. **d** FISH results showing the localization of *VIM-AS1* and IGF2BP1 (left) and *EPHA3* mRNA and IGF2BP1 (right). Bar graphs showing the relative colocalization fluorescence intensity of each FISH dataset. White arrows = colocalization region of *VIM-AS1* or *EPHA3* mRNA and IGF2BP1. Con Control, OE = *VIM-AS1* overexpression, KD = *VIM-AS1* knockdown, ND not detected, ***p < 0.001.

To further investigate how *VIM-AS1* influences the interaction between *EPHA3* mRNA and IGF2BP1, we established liver cells with increased or decreased *VIM-AS1* expression. Experimental validation revealed that *VIM-AS1* overexpression preferentially augmented the association of IGF2BP1 with *VIM-AS1*, concomitantly reducing its engagement with *EPHA3* mRNA (Fig. 5a). In contrast, a reduction in *VIM-AS1* levels diminished its interaction with IGF2BP1, thereby enhancing its binding to *EPHA3* mRNA (Fig. 5b). We hypothesized that the abundance of *VIM-AS1* may attenuate the affinity of IGF2BP1 for *EPHA3* mRNA, thereby diminishing the stability of *EPHA3* mRNA (Fig. 5c). Furthermore, *VIM-AS1* and IGF2BP1 colocalized following *VIM-AS1* overexpression, while the colocalization of *EPHA3* mRNA and IGF2BP1 was significantly abolished (Fig. 5d and Supplementary Fig. 5). These results indicated that *VIM-AS1* modulated *EPHA3* mRNA stability by altering IGF2BP1 binding dynamics.

### *EPHA3* mRNA was abundant in the fibroblasts associated with HCC

**Fig. 6:**
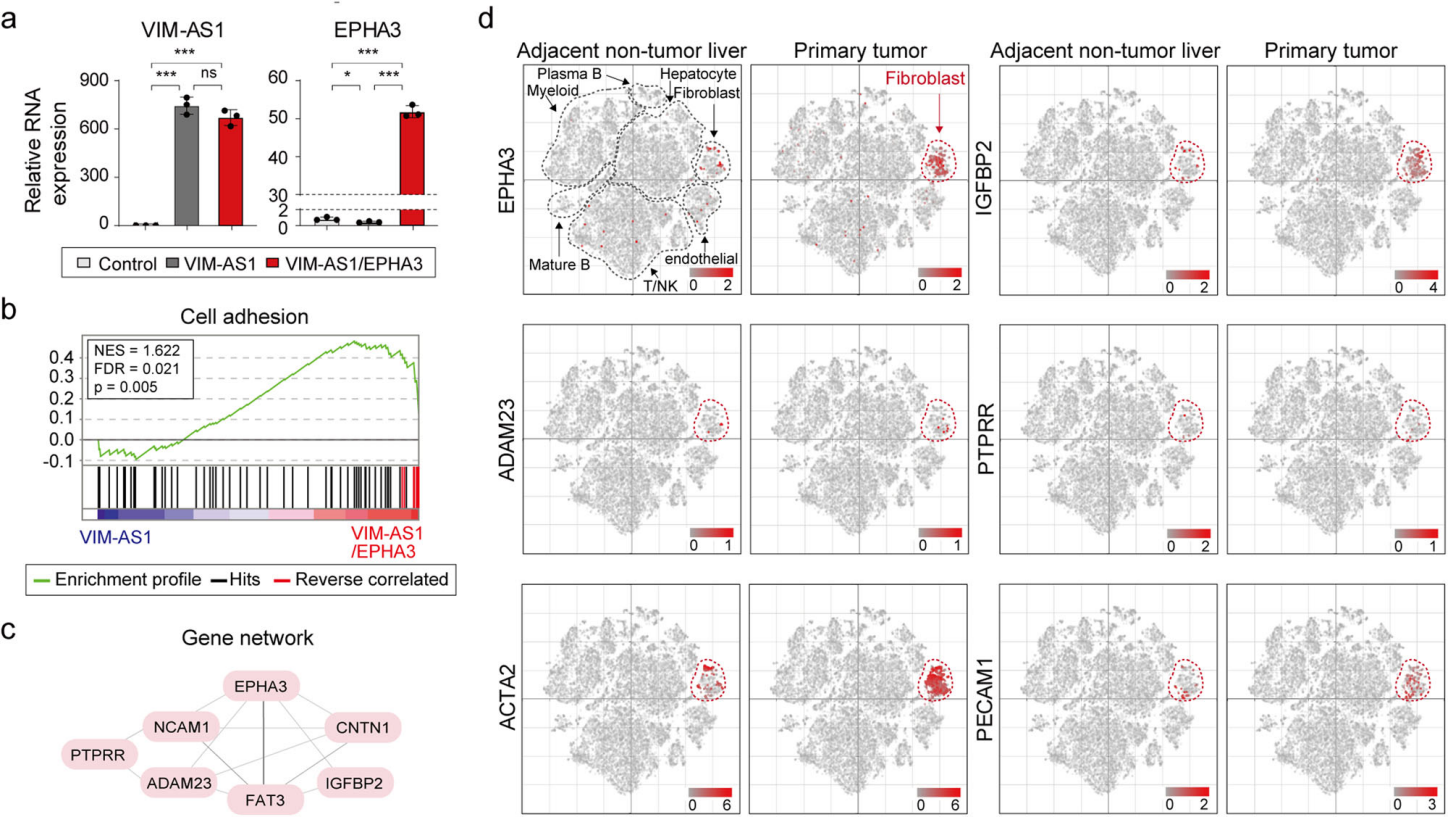
Genes regulated by the *VIM-AS1‒EPHA3* axis are involved in HCC aggressiveness. **a** Relative expression of *VIM-AS1* and *EPHA3* in *VIM-AS1-*overexpressing cells and *VIM-AS1* and *EPHA3* co-overexpressing cells. **b** GSEA indicates genes associated with cell adhesion. Red bars = DEGs whose expression was decreased by *VIM-AS1* overexpression but increased upon overexpression of both *VIM-AS1* and *EPHA3*. **c** Gene network analysis revealed that *EPHA3* mRNA, which is regulated by *VIM-AS1*, is downregulated/upregulated along with *EPHA3* mRNA expression. These data were analyzed via GENE NETWORK v2.0. **d** scRNA-seq profiles (GSE149614) of genes that are regulated along with *EPHA3* mRNA. *p < 0.05, ***p < 0.001, ns = not significant.

To determine the function of *EPHA3* mRNA regulation by *VIM-AS1* in HCC, we established *VIM-AS1*-overexpressing and *VIM-AS1-* and *EPHA3*-overexpressing cell lines (Fig. 6a) and performed RNA-seq analysis. The DEGs are listed in Supplementary Table 2. Interestingly, using GSEA, we confirmed that the expression of cell adhesion-related genes, whose expression was decreased by *VIM-AS1* overexpression, increased with the restoration of *EPHA3* mRNA (Fig. 6b). Furthermore, we confirmed the interconnections among these genes via gene network analysis (Fig. 6c), suggesting that they collectively modulated the malignancy of HCC via the *VIM-AS1‒EPHA3* regulatory axis.

Next, using single-cell RNA-seq analysis, we aimed to identify the subgroups of liver cancer cells in which the *VIM-AS1*–*EPHA3* regulatory axis altered gene expression (Fig. 6d). We observed that genes modulated via the *VIM-AS1‒EPHA3* axis, including *IGFBP2, ADAM23*, and *PTPRR*, presented markedly higher expression levels in HCC-associated fibroblasts than in control cells. Fibroblasts are present in tumors as cancer-associated fibroblasts (CAFs), which are involved in extracellular matrix deposition and remodeling, epithelial‒mesenchymal transition, and invasion. They typically express alpha-smooth muscle actin and contribute to tumor growth and angiogenesis^41–43^. Hence, we also examined the expression of *ACTA2* (α-SMA), a marker of CAFs, and *PECAM1* (CD31), a marker of vascular differentiation. As expected, the expression of these genes was elevated in HCC fibroblasts (Fig. 6d). These results indicate that genes regulated by *VIM-AS1*, including *EPHA3* mRNA, are involved in HCC aggressiveness in CAFs.

### Overexpression of *VIM-AS1* inhibited *EPHA3* mRNA and diminished tumorigenesis in vivo

**Fig. 7:**
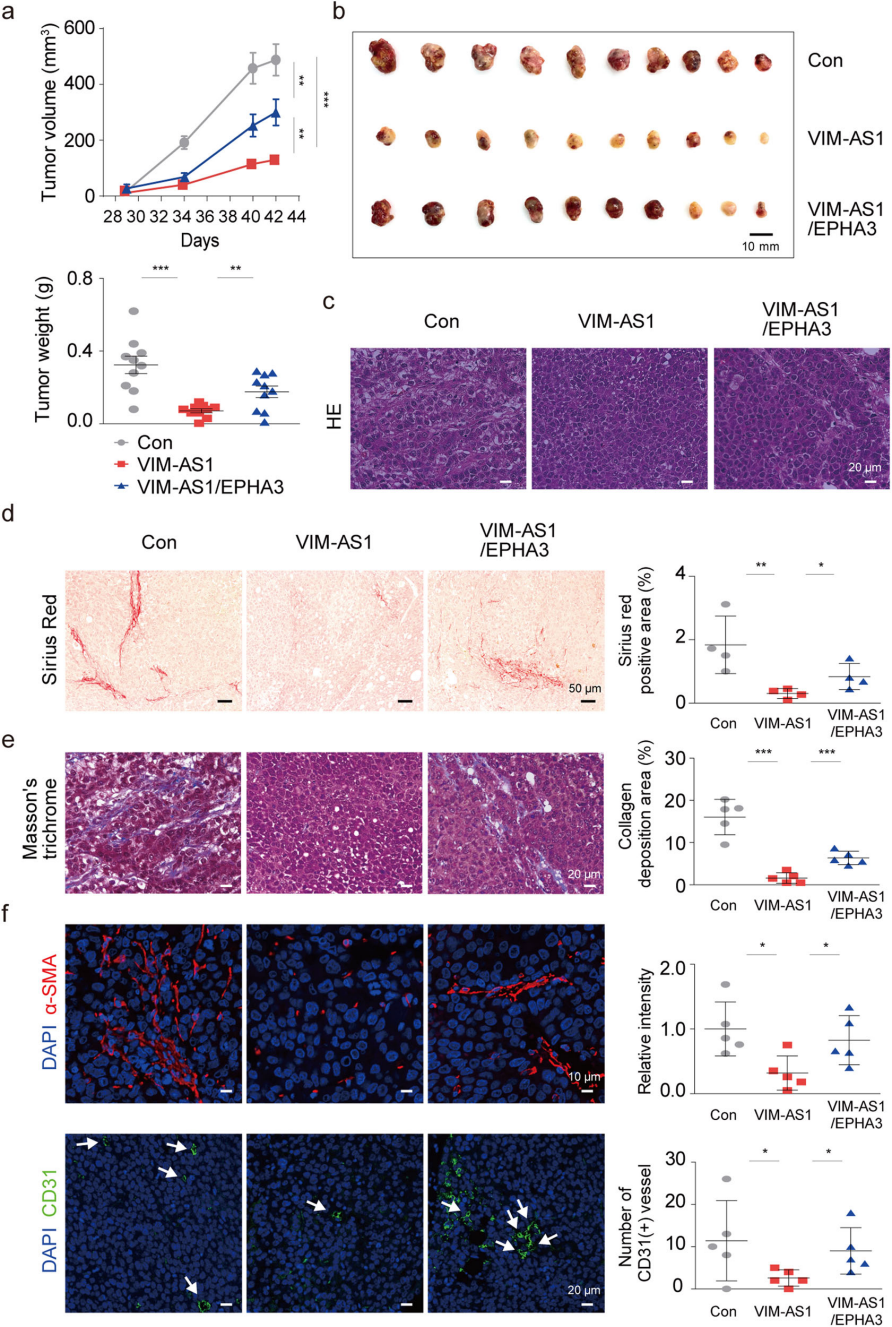
*VIM-AS1* inhibits tumor microenvironmental changes in HCC. **a** Graphs showing tumor volume and weight changes in each group. **b** Images of tumors resected from each group. **c** HE-stained sections showing cellular and structural changes. **d** Sirius Red staining of collagen deposits. **e** Masson’s trichrome staining with quantified areas of collagen deposition. **f** IF staining for α-SMA (top) and CD31 (bottom), with quantification. White arrows = CD31-positive vessels. Con = Control, *VIM-AS1* = *VIM-AS1* overexpression, *VIM-AS1/EPHA3* = *VIM-AS1* and *EPHA3* co-overexpression; *p < 0.05, **p < 0.01, ***p < 0.001.

To examine the effects of *EPHA3* mRNA regulation by *VIM-AS1* on tumorigenesis and the tumor microenvironment in vivo, *VIM-AS1*-overexpressing and *VIM-AS1-* and *EPHA3*-overexpressing cell lines were subcutaneously injected into nude mice, and tumor growth curves and weights were monitored. The tumor volume and weight in the *VIM-AS1*-overexpressing group were lower than those in the other groups, and *EPHA3* overexpression accelerated tumor growth in the *VIM-AS1*-overexpressing group (Fig. 7a, b). In addition, the EPHA3 protein level was lower in *VIM-AS1*-overexpressing tumors than in control tumors but was restored in *VIM-AS1-* and *EPHA3*-overexpressing tumors (Supplementary Fig. 6). These findings suggest that *VIM-AS1* regulates HCC tumorigenesis via EPHA3 in vivo. Hematoxylin and eosin (H&E) staining revealed abnormal nuclear and cytoplasmic morphologies in the control group, which was alleviated in the *VIM-AS1*-overexpressing group, and tissue deformation reappeared in the *VIM-AS1/EPHA3* co-overexpressing group (Fig. 7c). Sirius Red and Masson’s trichrome staining revealed many collagen fibers in the control group, which were reduced in the *VIM-AS1*-overexpressing group and increased in the *VIM-AS1/EPHA3*-co-overexpressing group (Fig. 7d, e). Immunofluorescence staining revealed that α-SMA expression and the number of CD31-positive vessels were significantly lower in the *VIM-AS1*-overexpressing group than in the control group. In addition, the number of α-SMA- and CD31-positive vessels was restored in the *VIM-AS1/EPHA3* co-overexpressing group (Fig. 7f). These results indicated that *VIM-AS1* exerts its antitumor effect by affecting CAFs and vessel formation and that *EPHA3* overexpression reversed these effects on CAFs and vessel formation in vivo.

## Discussion

In this study, we demonstrated that methylated cg02746869 was associated with a poor prognosis in HCC and that it directly regulated the expression of *VIM-AS1*, a noncoding RNA (Fig. 1). *VIM-AS1* regulated *EPHA3* mRNA stability in the cytoplasm, which required binding by the m6A reader IGF2BP1 (Figs. 3, 4 and 5). On the basis of these findings, we propose an epigenetic axis involving modifications of DNA methylation, lncRNA expression, and mRNA stability as a potential core panel for diagnosing and treating HCC.

Cancer is associated with various types of dysregulated events at the genomic, epigenomic, and transcriptional levels. Thus, the identification of interconnected aberrations at these levels may offer valuable evidence for understanding the progress in malignancy and developing prognostic/therapeutic targets for cancer. In this study, we systematically analyzed aberrations at various levels in the regulome to elucidate the malignant process of HCC. We showed that *VIM-AS1* expression was regulated by methylation in HCC. The enhancement and reduction of cg02746869 methylation via the CRISPR/dCas9 system not only changed the expression of target genes but also led to substantial alterations in the functional aspects of liver cancer cells, such as cell migration and invasion. In addition, by elucidating the alterations in histone modifications in this regulome, we demonstrated the mechanism underlying the progression of HCC, which included aberrations from the genomic to the transcriptional level. A series of experimental validations and transcriptome analyses revealed the biological and clinical implications of the *VIM-AS1*–*EPHA3* axis in HCC progression. Notably, the methylation status of cg02746869 in *VIM-AS1* is the master regulator of the *VIM-AS1*–*EPHA3* axis, indicating that cg02746869–*VIM-AS1*–*EPHA3* acts as the pathogenic circuit of HCC aggressiveness.

Several studies have reported a close association between *VIM-AS1* and the pathophysiology of various diseases, such as gastric cancer, colon cancer, and preeclampsia. One study revealed that high expression of *VIM-AS1* in human gastric cancer tissues was correlated with TNM stage. Knocking down *VIM-AS1* inhibited the migration and invasion of gastric cancer cells by suppressing the Wnt/β-catenin signaling pathway^44^ through downregulating FZD1. In colon cancer, *VIM-AS1* upregulation is correlated with cancer progression and poor survival, whereas *VIM-AS1* downregulation suppresses the EMT and migration of colon cancer cells^45^. Overall, high expression of *VIM-AS1* in gastric and colon cancer tissues facilitated cell migration and possibly metastasis by regulating the EMT process. *VIM-AS1* has also been implicated in the progression of other diseases, such as preeclampsia, a pregnancy-specific condition characterized by hypertension and proteinuria. In preeclampsia, a gradual decrease in *VIM-AS1* levels is associated with the disease’s progression. Moreover, supplementation with *VIM-AS1* enhanced the diminished EMT capacity of hypoxia-induced placental trophoblast cells^46^. In human liver cancer, *VIM-AS1* was found to be downregulated, leading to increased tumor aggressiveness, including the migration and invasion of tumor cells, through the regulation of *EPHA3* mRNA stability. Overexpression of *VIM-AS1* mitigated the tumor malignancy and tumorigenesis of HCC both in vitro and in vivo. Interestingly, in contrast to previous studies in gastric and colorectal cancers, where *VIM-AS1* expression was increased and linked to increased cell migration, our study in HCC patients revealed decreased expression of *VIM-AS1*, which was associated with increased cell migration and invasion. These results suggest that the tissue- and disease-specific expression patterns of *VIM-AS1* could serve as potential biomarkers and play significant roles in diverse tissues.

A phase I clinical trial was conducted targeting glioblastoma patients with a drug (NCT03374943) that regulates the protein level of EPHA3, which we identified as the final player in the epigenetic regulome associated with the aggressiveness of HCC. Upregulation of *EPHA3* mRNA in the tumor-initiating cell population of gliomas and in tumor cells maintained in an undifferentiated state contributed to their tumorigenic potential; this was evident from the reduction in tumorigenic potential upon attenuation of the EPHA3 protein^47^. A phase I clinical trial evaluating the anti-EPHA3 monoclonal antibody ifabotuzumab in patients with recurrent glioblastoma demonstrated its efficacy in targeting the tumor microenvironment while sparing normal tissues^48,49^. Elevated protein levels of EPHA3 are associated with aggressive tumor characteristics and an unfavorable prognosis in HCC. In addition, downregulation of *EPHA3* mRNA mitigates HCC cell invasiveness via the modulation of VEGF signaling pathways^50^; nonetheless, therapeutic interventions targeting EPHA3 have not been attempted. In this study, we identified an epigenetic modulator capable of regulating the expression levels of *EPHA3* mRNA. We confirmed that modulating the abundance of *VIM-AS1* via alterations in the levels of DNA methylation (Fig. 1) affected the stability and expression of *EPHA3* mRNA (Figs. 3 and 5). Our findings will contribute to the development of therapeutic strategies targeting EPHA3 to mitigate the aggressiveness of HCC, leveraging the ability to quantitatively detect alterations in *VIM-AS1* expression and changes in DNA methylation levels at the *VIM-AS1* locus.

Overall, we revealed that the tumor-suppressive function of *VIM-AS1* was regulated by cg02746869, which modulated *EPHA3* mRNA stability, suggesting a basis for HCC progression and the development of potential biomarkers and therapeutic targets for HCC.

## Supplementary information

Supplementary Information

## Data Availability

All relevant data in this study are available from the corresponding authors upon reasonable request.
