# Supplementary material for: VIM-AS1, which is regulated by CpG methylation, cooperates with IGF2BP1 to inhibit tumor aggressiveness via EPHA3 degradation in hepatocellular carcinoma
Source: Exp Mol Med. 2024 Dec 2;56(12):2617–30. doi: 10.1038/s12276-024-01352-6 (PMC11671536; doi:10.1038/s12276-024-01352-6)
Supplement: Supplementary file 1 — Supplementary Information [file 12276_2024_1352_MOESM1_ESM.pdf]

Supplementary Information

Supplementary Figures

Supplementary Fig. 1

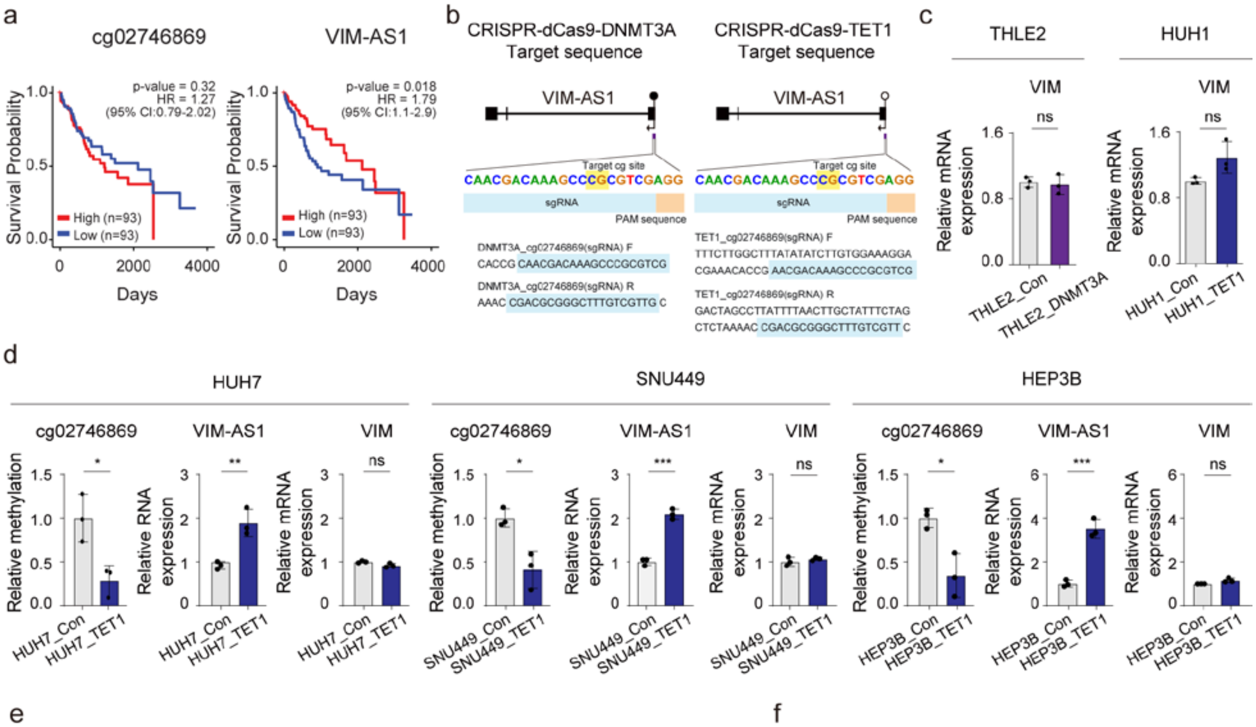

Screening	Motifs	Name	P-value
30bp	CAAGGTCA	SF1(NR)	1e-2
40bp	TCAAGGTCA	Nr5a2(NR)	1e-2
	ATTTCCTG	EWS(ETS)	1e-2
	TGACCT	RARα(NR)	1e-2
50bp	CAAGGTCA	SF1(NR)	1e-2
	TCAAGGTCA	Nr5a2(NR)	1e-2

Supplementary Fig. 1. Altered cg02746869 methylation and *VIM-AS1* expression affected overall survival in patient with HCC.

a Kaplan–Meier survival curves representing the impact of methylation level of cg02746869 (left) and *VIM-AS1* expression levels (right) in patients with TCGA-LIHC. These data were analyzed using TCGA-LIHC upper and lower quartile values. **b** Schematic illustration represents the gRNA for targeting cg02746869 region and each primer sequence for gRNA synthesis. **c** The relative mRNA expression of *VIM* in THLE2 and HUH1 with modified methylation of cg02746869. **d** The relative cg02746869 methylation, *VIM-AS1* expression and *VIM* mRNA expression in various cancer cells including HUH7, SNU449, and HEP3B. **e** Density plot showing the enrichment of ChIP-seq peaks of H3K4me3 and H3K27ac near the 50 CpG sites and near all TSSs in HUH1 and THLE2. **f** Predicted transcription factors that may bind to 50 CpG sites found using motif search analysis. *p < 0.05, **p < 0.01, ***p < 0.001, ns = not significant.

Supplementary Fig. 2

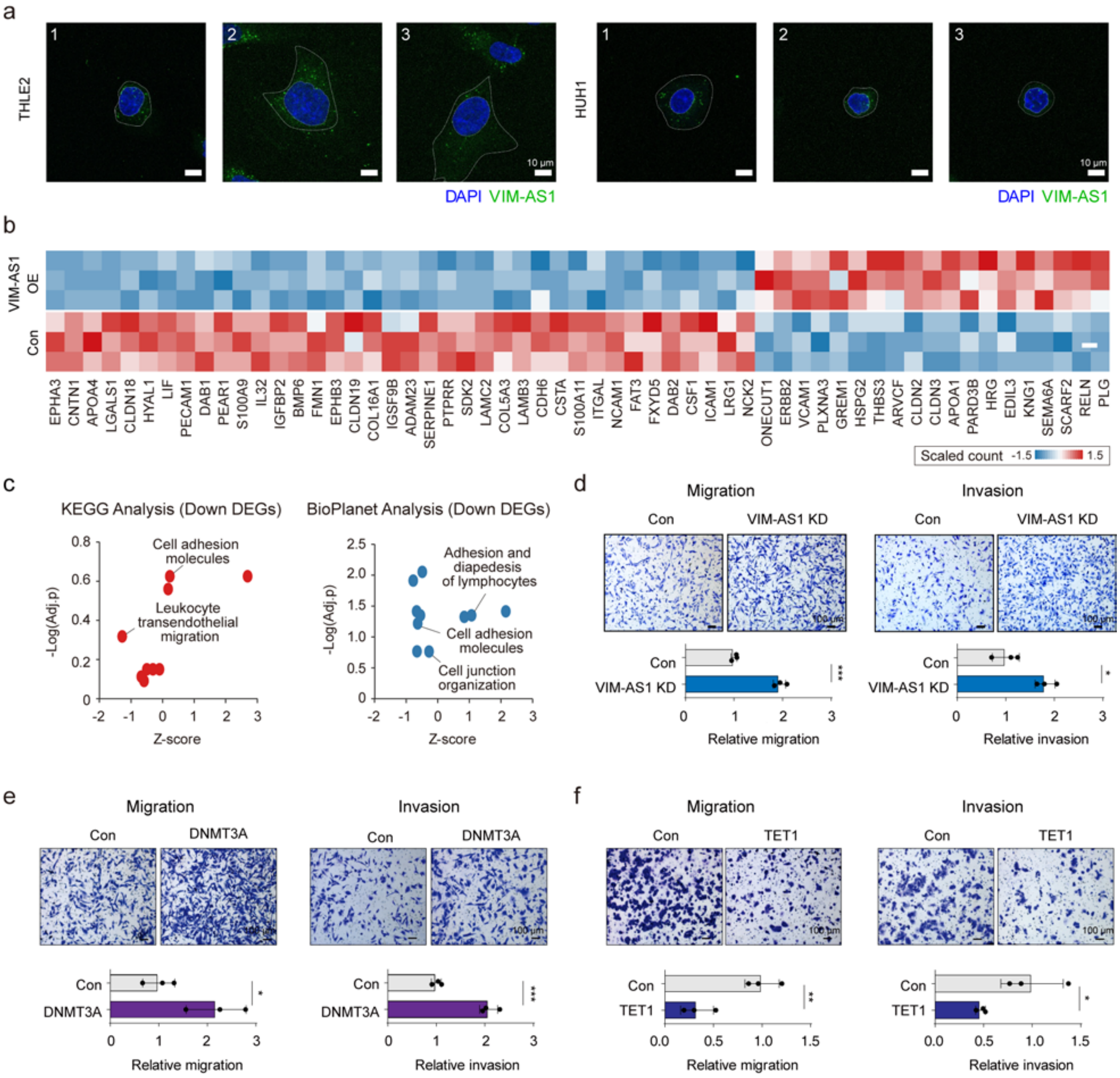

Supplementary Fig. 2. ***VIM-AS1* regulates genes associated with cell adhesion.**

a Images of *VIM-AS1* localization was detected using FISH in THLE2 and HUH1. **b** Heatmap of down-regulated DEGs associated with cell adhesion. **c** Dot plot representing the result of ontology analysis for DEGs downregulated by the enriched *VIM-AS1*. **d** Transwell migration and invasion assays following *VIM-AS1* knockdown (si*VIM-AS1*) compared those in the control (siCon). Assays for migration and invasion after DNMT3A-induced methylation (**e**) and TET1-induced demethylation (**f**). Con = empty vector, DNMT3A = hypermethylated cg02746869, TET1 = hypomethylated cg02746869. *p < 0.05, **p < 0.01, ***p < 0.001.

Supplementary Fig. 3

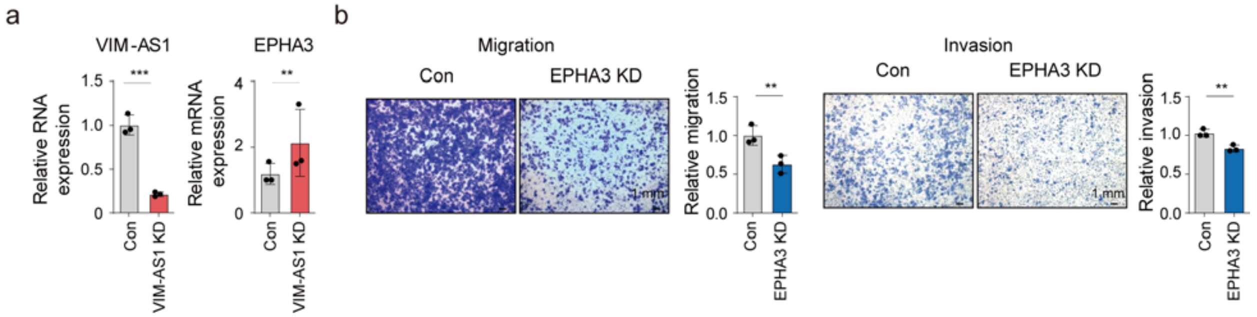

Supplementary Fig. 3. Decreased *EPHA3*, regulated by *VIM-AS1*, attenuated migration and invasion of HCC cells a Relative expression levels of *VIM-AS1* and *EPHA3* in THLE2 cells after *VIM-AS1* knockdown (*VIM-AS1* KD*)* **b** Representative images and quantitative analysis of the migration and invasion assays in HUH7 cells after *EPHA3* knockdown (*EPHA3* KD). Con = siControl, **p < 0.01, ***p < 0.001.

Supplementary Fig. 4

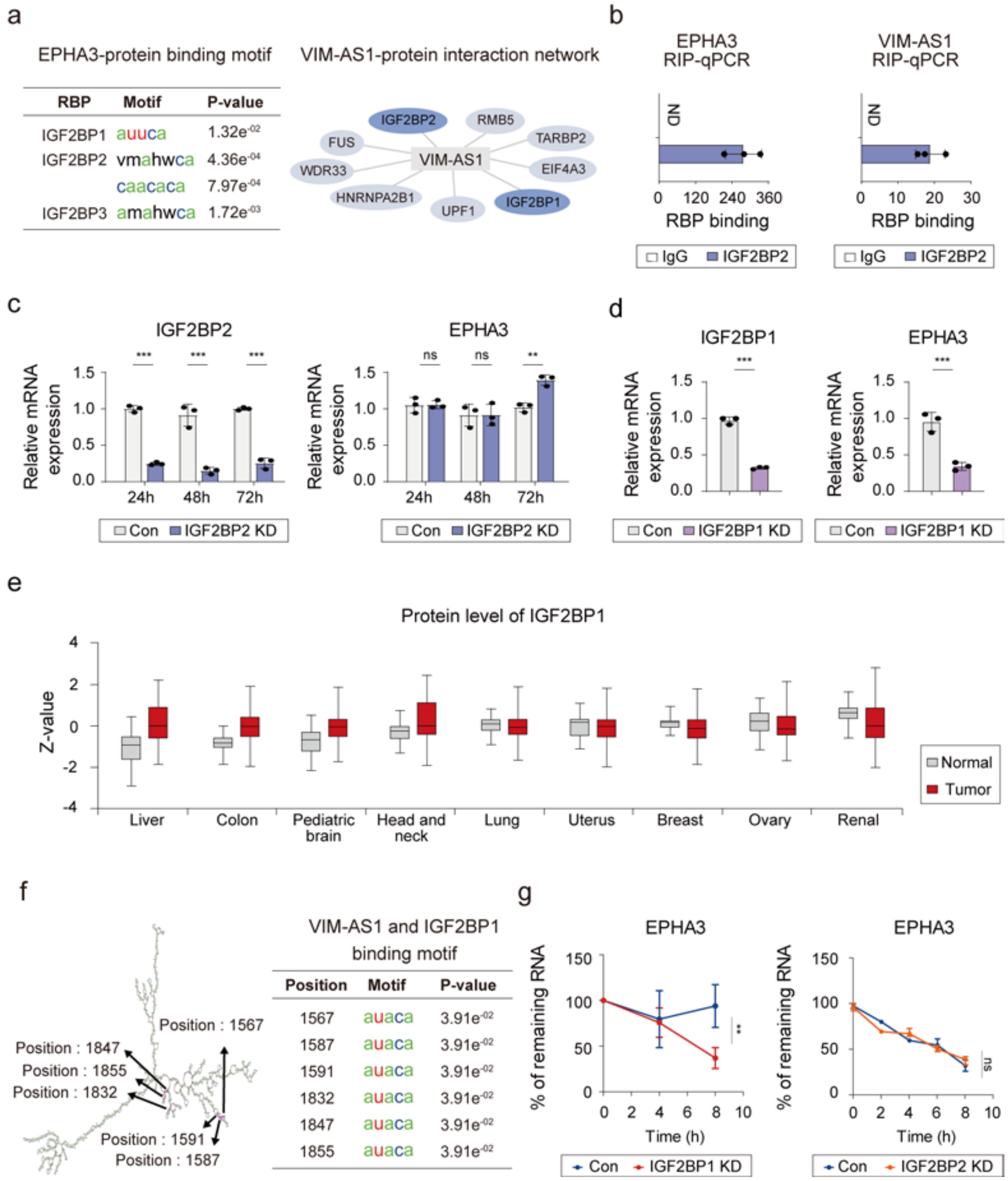

Supplementary Fig. 4. IGF2BP1 potentially binds to *VIM-AS1*, regulating *EPHA3* mRNA stability. a Motifs identified within the *EPHA3* mRNA recognized by IGF2BP1/2/3 using RBPmap (left). The interaction network of *VIM-AS1* analyzed using CLING (right). **b** RIP-qPCR assays showing *EPHA3* and *VIM-AS1* RNA bound to IGF2BP2 in HUH1. **c** Effect of IGF2BP2 silencing on *EPHA3* mRNA expression. **d** Relative mRNA expression levels in HUH7 cells after knockdown of IGF2BP1. **e** Boxplot showing the differential protein levels of IGF2BP1 in various cancers using UALCAN. **f** Structure of *VIM-AS1* with IGF2BP1 binding site predicted using ViennaRNA Web Services. Pink highlight and black arrow: IGF2BP1 binding region predicted using RBP map in *VIM-AS1*. **g** Stability curve of *EPHA3* mRNA after actinomycin D treatment in

IGF2BP1 siRNA or *IGF2BP2* siRNA transfected HUH1 cells. Con = control, *IGF2BP1* KD = *IGF2BP1* knockdown. *IGF2BP2* KD = *IGF2BP2* knockdown. **$p < 0.01$, ***$p < 0.001$, ns = not significant.

Supplementary Fig. 5

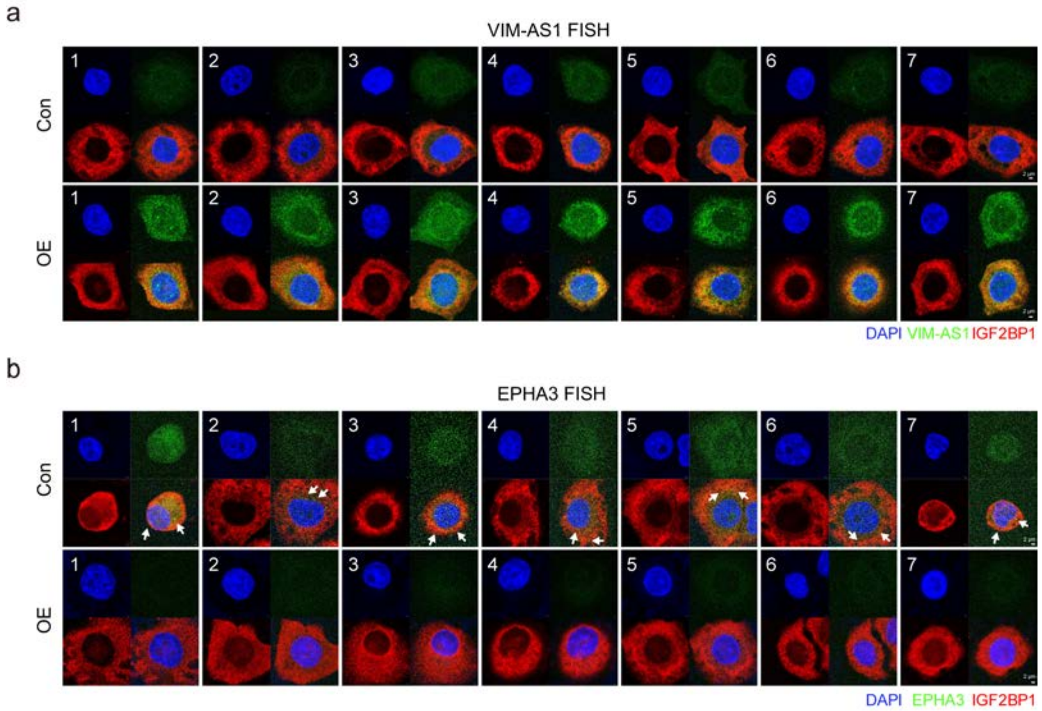

Supplementary Fig. 5. Overexpression of *VIM-AS1* regulates interaction between IGF2BP1 and *EPHA3* mRNA. The FISH images represent the co-localization of IGF2BP1 with *VIM-AS1* **(a)** and *EPHA3* mRNA **(b)**. White arrows = co-localization region of *EPHA3* mRNA and IGF2BP1.

Supplementary Fig. 6

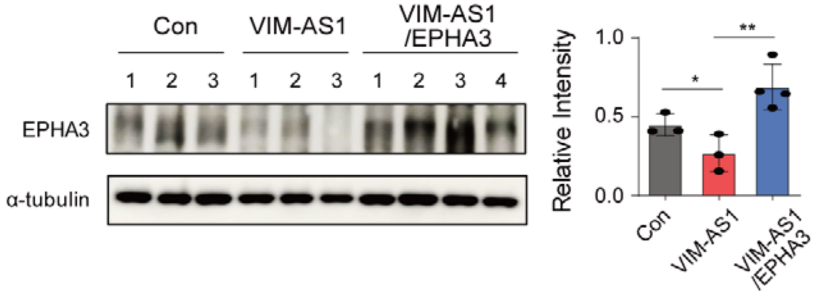

Supplementary Fig. 6. EPHA3 protein level in HCC xenograft tumors. An immunoblot of EPHA3 and α-tubulin in HCC xenograft samples (left). α-tubulin used as loading control. The bar graph shows the band intensity of EPHA3 normalized to α-tubulin (right). *$p < 0.05$, **$p < 0.01$.

Supplementary Table 1. List of genes differentially expressed after *VIM-AS1* overexpression.

[Down-regulated genes]

Gene Name	CON1	CON2	CON3	VIM-AS1 OE1	VIM-AS1 OE2	VIM-AS1 OE3
A4GALT	4.7	2.6	8.4	1.1	1.1	0.0
ABCA4	1948.0	2067.9	2093.0	1382.6	1498.0	1475.7
ACP5	104.5	120.6	126.7	80.1	73.5	106.8
ADAM23	104.5	106.5	73.2	47.4	63.9	60.7
ADAMTS10	61.7	32.6	58.2	30.5	35.1	28.3
AGAP2-AS1	69.3	79.2	78.8	42.9	58.6	57.6
AIM1L	71.2	76.6	72.3	49.7	44.7	46.1
AKR1B1	4799.3	4609.4	4873.9	3431.2	3156.9	3034.2
ALOX12P2	94.0	85.4	110.7	67.7	79.9	65.9
ANKRD1	30.4	17.6	27.2	13.5	14.9	7.3
ANXA3	179.5	150.5	160.5	134.3	106.5	100.5
APOA4	16.1	25.5	16.9	5.6	3.2	5.2
AQP7	20.9	19.4	23.5	6.8	5.3	13.6
ASAH2	57.0	61.6	47.9	36.1	35.1	35.6
ATP5I	443.6	467.5	547.2	349.9	366.3	329.7
BBOX1	332.4	312.5	308.8	193.0	198.0	163.3
BMP6	277.3	258.8	281.6	144.5	164.0	148.6
C10orf10	143.4	133.8	127.6	79.0	63.9	71.2
C19orf33	587.0	544.9	673.9	453.7	448.2	438.5
C1R	515.7	559.0	611.9	373.6	406.7	406.1
C1RL-AS1	76.9	56.3	72.3	36.1	44.7	44.0
C1orf220	80.7	102.1	109.8	65.5	54.3	75.4
C4BPB	150.1	153.2	141.7	98.2	108.6	113.0
CAPN9	316.3	335.4	317.2	223.5	211.9	213.5
CCDC183-AS1	123.5	125.0	153.0	91.4	73.5	70.1
CCL15	593.6	544.9	590.3	461.6	468.5	387.3
CDA	150.1	150.5	168.0	112.9	101.1	101.5
CDCP1	19.0	24.6	22.5	10.2	8.5	9.4
CDH6	48.4	57.2	54.4	44.0	34.1	27.2
CDKN1A	211.8	197.2	241.2	141.1	134.2	134.0
CEMIP	122.5	92.4	116.4	68.8	76.7	60.7

CHAD	38.0	33.5	46.9	16.9	27.7	18.8
CKMT1B	49.4	21.1	15.0	4.5	13.8	12.6
CLDN18	7.6	10.6	13.1	1.1	3.2	2.1
CLDN19	5.7	2.6	9.4	1.1	0.0	1.0
CNKSR2	59.8	53.7	54.4	29.3	38.3	32.4
CNTN1	128.2	120.6	120.1	39.5	34.1	38.7
COL16A1	39.9	46.7	50.7	18.1	31.9	25.1
COL5A3	81.7	99.5	104.2	51.9	64.9	70.1
CSF1	756.0	730.7	817.5	632.1	563.2	517.0
CSTA	96.9	110.0	120.1	73.4	73.5	76.4
CT55	22.8	19.4	22.5	12.4	10.6	9.4
CTAGE15	98.8	107.4	114.5	62.1	88.4	48.1
CTSL	3750.7	3892.0	4261.0	2689.6	2686.3	2633.3
CYP24A1	1092.3	1130.4	1150.7	852.2	830.5	562.0
CYP2B7P	600.3	569.6	645.7	344.2	408.8	460.5
CYP2C9	51.3	35.2	55.4	33.9	23.4	27.2
DAB1	26.6	21.1	19.7	5.6	5.3	12.6
DAB2	367.6	361.8	350.1	270.9	267.2	259.6
DCAF8L2	25.6	34.3	25.3	9.0	10.6	13.6
DGKG	148.2	125.0	114.5	84.7	102.2	90.0
DIO2	156.7	140.9	121.1	54.2	51.1	34.5
DPM3	141.5	140.0	124.8	110.6	72.4	114.1
DUSP5	203.3	187.5	194.3	130.9	115.0	126.6
DUSP6	414.1	420.8	458.9	285.6	338.6	328.6
EGFR-AS1	46.5	39.6	39.4	15.8	19.2	20.9
EGLN3	179.5	174.3	211.2	106.1	117.1	96.3
EMP1	247.9	225.4	269.4	158.0	167.2	115.1
EPHA3	29.4	35.2	20.6	2.3	3.2	2.1
EPHB3	259.3	304.6	297.5	151.2	177.8	165.4
EPHB6	359.0	328.4	376.4	117.4	96.9	100.5
ERC2	32.3	31.7	40.4	10.2	11.7	12.6
FAM173B	191.9	154.9	178.3	126.4	146.9	127.7
FAM174B	209.9	210.4	201.8	159.1	146.9	158.0
FAT3	107.3	89.8	94.8	72.2	67.1	72.2
FBXO2	30.4	22.9	26.3	11.3	9.6	14.7
FGFR1	343.8	309.9	396.1	197.5	238.5	211.4

FHDC1	231.7	221.8	219.6	171.6	174.6	166.4
FLVCR1-AS1	205.2	196.3	205.5	130.9	161.8	152.8
FMN1	24.7	26.4	17.8	7.9	13.8	13.6
FMO9P	43.7	59.0	55.4	36.1	26.6	37.7
FRMD3	80.7	107.4	94.8	51.9	58.6	58.6
FSCN1	136.8	146.1	138.9	123.0	111.8	82.7
FXYD5	145.3	141.7	169.9	115.1	119.2	102.6
G0S2	509.1	581.9	596.9	349.9	350.3	338.1
GATA2	336.2	324.0	382.0	234.8	240.6	265.8
GLIPR1	371.4	360.1	367.0	311.5	256.6	214.6
GLRX	455.9	480.7	474.0	303.6	338.6	353.8
GLYATL1	26.6	35.2	31.0	6.8	19.2	19.9
GNG2	23.7	15.8	19.7	2.3	10.6	12.6
GPRC5A	44.6	33.5	47.9	20.3	25.6	18.8
H19	76.0	66.0	117.3	30.5	60.7	45.0
HAL	171.0	155.8	172.7	95.9	104.3	112.0
HDDC3	179.5	172.5	195.2	140.0	139.5	137.1
HECW2	180.5	175.2	174.6	97.1	94.8	80.6
HHIPL2	834.9	888.3	911.3	621.9	655.9	687.6
HIST1H2AC	135.8	102.1	147.4	93.7	73.5	100.5
HMOX1	3151.4	3309.2	3289.6	1766.4	1936.7	1780.3
HP	228.0	251.8	254.3	136.6	131.0	167.5
HPD	4479.2	4554.0	4782.8	2767.5	3014.2	2896.0
HRCT1	159.6	119.7	146.4	101.6	111.8	87.9
HSD17B6	73.1	57.2	75.1	40.6	40.5	52.3
HSPB8	171.0	173.4	151.1	55.3	44.7	38.7
HYAL1	46.5	59.9	56.3	28.2	18.1	25.1
ICAM1	268.8	274.7	327.6	217.8	217.2	212.5
IFI27L2	51.3	60.7	61.9	37.2	43.7	37.7
IGFBP2	127.3	118.8	138.9	65.5	76.7	68.0
IGSF9B	7.6	7.9	1.9	0.0	1.1	1.0
IL32	432.2	387.3	372.6	234.8	221.5	185.3
INHBA	57.9	44.9	64.8	33.9	28.7	30.4
ITGAL	117.8	120.6	130.5	73.4	89.4	97.3
ITM2A	162.4	176.1	164.2	125.3	117.1	115.1
JAKMIP1	11.4	12.3	10.3	3.4	4.3	5.2

KCNH7	158.6	137.3	125.8	103.8	94.8	105.7
KIF21B	282.1	213.0	237.5	179.5	181.0	153.9
KIF5A	56.0	51.9	61.9	36.1	27.7	46.1
KLRC2	71.2	60.7	63.8	38.4	51.1	36.6
KRT19	1511.1	1560.0	1711.0	1144.5	1190.3	913.7
KRT81	453.1	457.8	499.3	367.9	296.0	157.0
LAMB3	455.9	454.3	529.3	295.7	348.2	325.5
LAMC2	74.1	72.2	74.1	48.5	47.9	45.0
LGALS1	422.7	413.8	509.6	190.7	157.6	165.4
LIF	72.2	78.3	83.5	30.5	25.6	42.9
LINC00969	40.8	59.0	47.9	32.7	21.3	36.6
LINC01271	30.4	39.6	42.2	14.7	22.4	24.1
LINC01370	36.1	21.1	18.8	5.6	4.3	10.5
LINC01559	15.2	7.9	21.6	4.5	2.1	4.2
LINC01588	31.3	28.2	27.2	12.4	17.0	12.6
LOC100419583	77.9	66.0	78.8	51.9	52.2	55.5
LOC100505549	24.7	24.6	28.2	11.3	17.0	11.5
LOC100506100	86.4	72.2	46.0	46.3	45.8	38.7
LOC101927934	75.0	57.2	69.5	58.7	35.1	42.9
LOC103091866	29.4	30.8	29.1	19.2	19.2	9.4
LOC151174	1.9	12.3	11.3	1.1	1.1	2.1
LOC283335	152.0	168.1	181.1	105.0	143.7	119.3
LOC642852	131.1	152.3	142.7	112.9	91.6	108.8
LOH12CR2	12.3	7.0	8.4	3.4	2.1	3.1
LOX	66.5	60.7	59.1	29.3	43.7	30.4
LRG1	315.3	344.2	286.3	224.6	251.3	228.2
LUCAT1	345.7	337.2	361.3	221.2	258.7	283.6
LUM	282.1	290.5	237.5	177.2	168.2	182.1
MAB21L3	106.4	88.9	80.7	49.7	39.4	49.2
MRO	65.5	93.3	75.1	60.9	42.6	53.4
MUC3A	95.9	104.8	111.7	71.1	82.0	78.5
MVP	1278.4	1202.5	1410.6	1019.2	930.6	997.4
MYH16	26.6	22.9	28.2	14.7	7.5	7.3
NAA11	36.1	28.2	28.2	10.2	14.9	16.7
NAALADL1	66.5	51.9	113.6	33.9	53.2	59.7
NAV3	227.0	215.7	224.3	195.3	149.1	162.2

NCAM1	1024.8	1078.4	1041.8	749.4	750.6	735.8
NCK2	541.4	485.1	528.4	440.2	390.7	351.7
NCR3LG1	68.4	76.6	48.8	42.9	35.1	33.5
NDUFB1	268.8	305.5	352.9	221.2	224.7	240.7
NEBL	52.2	55.5	63.8	28.2	43.7	40.8
NNMT	21.8	19.4	20.6	3.4	3.2	8.4
NOSTRIN	174.8	189.3	176.4	140.0	134.2	129.8
NOTCH3	930.8	922.6	1085.9	670.4	697.4	740.0
NPR2	446.4	427.0	459.9	303.6	333.3	370.5
NRG4	152.0	132.1	124.8	110.6	105.4	91.1
NSUN3	288.7	322.2	291.9	211.1	230.0	240.7
NUTM2A-AS1	110.2	118.0	124.8	99.3	85.2	84.8
OASL	76.9	79.2	82.6	48.5	64.9	56.5
OR51B5	90.2	76.6	68.5	59.8	50.0	48.1
OXSM	247.9	248.3	211.2	180.6	177.8	174.8
PALM3	51.3	34.3	64.8	23.7	28.7	29.3
PAQR5	290.6	334.5	269.4	244.9	210.8	179.0
PART1	107.3	126.8	135.2	71.1	66.0	50.2
PCBP1-AS1	13.3	10.6	9.4	3.4	2.1	5.2
PEAR1	25.6	27.3	31.9	13.5	6.4	14.7
PECAM1	29.4	32.6	30.0	12.4	16.0	15.7
PHKA1	340.0	322.2	352.0	240.4	257.7	222.9
PITPNA-AS1	74.1	75.7	107.0	65.5	56.4	57.6
PLAC8	54.1	52.8	46.0	21.4	38.3	34.5
PRAP1	2089.5	2193.8	2581.9	1643.4	1775.9	1810.7
PRRT3-AS1	95.9	84.5	90.1	65.5	62.8	65.9
PSMB9	128.2	129.4	106.1	97.1	100.1	74.3
PTPRR	110.2	106.5	93.9	67.7	72.4	54.4
RAET1G	8.5	6.2	8.4	2.3	2.1	1.0
RDH16	20.9	29.9	37.5	19.2	12.8	15.7
RPL31P11	21.8	19.4	15.0	6.8	7.5	8.4
S100A11	4645.4	4483.5	4898.3	3316.1	3415.6	2977.6
S100A6	18697.6	18687.7	21206.4	15408.7	15428.7	14087.5
S100A9	75.0	87.2	74.1	32.7	41.5	47.1
S1PR3	121.6	86.3	107.9	84.7	73.5	76.4
SAMD9	240.3	202.5	229.9	179.5	160.8	130.8

SAMD9L	153.9	150.5	108.9	94.8	102.2	87.9
SCN9A	77.9	73.1	53.5	29.3	24.5	27.2
SCO2	141.5	162.0	155.8	117.4	117.1	108.8
SDHAF3	373.3	352.1	352.9	258.5	256.6	259.6
SDK2	59.8	50.2	48.8	33.9	34.1	33.5
SEC14L4	63.6	51.9	58.2	28.2	31.9	46.1
SERPINE1	2457.1	2553.9	2804.4	1535.0	1657.8	1552.1
SETBP1	100.7	80.1	93.9	60.9	54.3	68.0
SFN	2027.8	2033.6	2192.4	1627.6	1659.9	1496.7
SFRP4	157.7	157.6	148.3	130.9	101.1	90.0
SH2D5	92.1	85.4	86.3	37.2	53.2	50.2
SH3BGRL3	1495.0	1480.7	1521.4	1137.7	1230.8	950.3
SH3BP1	146.3	143.5	169.9	109.5	116.1	120.4
SLC19A3	118.7	90.7	122.9	63.2	66.0	46.1
SLC22A11	82.6	88.9	103.2	66.6	66.0	51.3
SLC23A3	139.6	131.2	172.7	109.5	80.9	95.2
SLC25A12	224.2	199.8	202.7	164.8	145.9	149.7
SLC51B	51.3	60.7	46.0	16.9	34.1	29.3
SLC7A7	245.0	286.1	246.8	185.1	159.7	153.9
SLC9A2	398.0	404.1	343.5	258.5	263.0	318.2
SLPI	143.4	132.9	122.9	105.0	94.8	93.1
SMIM4	166.2	143.5	150.2	100.5	110.7	132.9
SNHG10	107.3	109.2	122.0	85.8	92.6	81.6
SPDYE6	27.5	15.0	27.2	13.5	4.3	12.6
SSUH2	32.3	36.1	51.6	24.8	18.1	29.3
ST3GAL5	163.4	139.1	135.2	107.2	93.7	112.0
SULT1A3	375.2	377.7	396.1	273.1	263.0	345.4
SULT1A4	375.2	377.7	396.1	273.1	263.0	345.4
TACR1	281.1	239.5	248.7	215.6	195.9	177.9
TCN2	139.6	138.2	187.7	120.8	119.2	113.0
TLDC2	125.4	124.1	137.0	94.8	99.0	95.2
TM4SF19	302.0	282.6	334.1	230.3	239.6	217.7
TMC6	86.4	86.3	87.3	47.4	75.6	54.4
TMEM176B	888.1	1005.3	1004.2	744.9	696.3	776.6
TMEM216	108.3	99.5	96.7	58.7	93.7	71.2
TMEM53	104.5	84.5	102.3	64.3	74.5	71.2

TMEM99	102.6	92.4	105.1	67.7	60.7	55.5
TMPRSS3	11.4	15.8	13.1	5.6	3.2	3.1
TPK1	48.4	51.1	52.6	37.2	35.1	29.3
TREX1	75.0	69.5	69.5	39.5	37.3	45.0
TRPM2	58.9	43.1	66.6	41.8	35.1	32.4
TSPAN18	43.7	55.5	72.3	18.1	21.3	16.7
TSPEAR-AS1	17.1	12.3	10.3	2.3	1.1	2.1
TSTD1	160.5	142.6	153.0	111.7	120.3	108.8
TTLL7	190.0	181.3	195.2	144.5	139.5	150.7
TUBB3	76.9	76.6	62.9	47.4	41.5	17.8
TUBB6	130.1	124.1	107.9	91.4	67.1	69.1
UBD	72.2	100.4	90.1	48.5	44.7	53.4
UPB1	133.9	148.8	156.7	106.1	105.4	109.9
UPP1	190.0	226.2	224.3	176.1	178.9	134.0
UQCRHL	159.6	114.4	176.4	94.8	119.2	129.8
VIL1	4162.0	4149.0	4375.5	3190.8	3233.5	3247.7
VSX1	11.4	25.5	18.8	7.9	11.7	6.3
VWA1	73.1	86.3	79.8	60.9	35.1	62.8
ZBED6CL	832.0	750.9	854.1	549.7	483.4	657.3
ZNF655	34.2	37.9	37.5	26.0	16.0	13.6

[Up-regulated genes]

Gene Name	CON1	CON2	CON3	VIM-AS1 OE1	VIM-AS1 OE2	VIM-AS1 OE3
AASS	28.5	34.3	35.7	44.0	52.2	56.5
ABCD1	47.5	57.2	54.4	82.4	80.9	62.8
ACTL8	112.1	119.7	116.4	225.7	245.9	194.7
ADH1A	59.8	81.0	55.4	232.5	209.7	260.6
ADH1B	93.1	93.3	76.0	203.2	197.0	215.6
AFM	57.0	34.3	51.6	71.1	77.7	82.7
ALDOB	285.9	310.8	319.1	458.2	450.4	489.8
ANTXR2	77.9	64.3	64.8	83.5	103.3	105.7
ANXA6	5.7	4.4	4.7	11.3	14.9	11.5
APOA1	1175.8	1242.2	1299.9	1673.8	1870.7	1938.3
ARHGAP33	75.0	83.6	107.9	151.2	119.2	103.6
ARSG	14.2	16.7	17.8	28.2	31.9	29.3

ARVCF	54.1	52.8	61.9	65.5	83.0	90.0
ATF3	105.4	99.5	92.0	127.5	141.6	118.3
ATG16L2	36.1	56.3	53.5	68.8	84.1	93.1
ATP6V0E2	102.6	131.2	116.4	167.0	159.7	158.0
B3GALT6	185.2	196.3	229.9	261.9	282.1	252.2
BAHCC1	228.9	219.2	229.0	338.6	363.1	417.6
BCHE	280.2	274.7	244.0	362.3	379.0	458.4
BEX2	0.9	3.5	0.9	4.5	5.3	12.6
BFSP1	29.4	33.5	32.8	64.3	57.5	48.1
C9orf69	798.8	800.2	960.1	1233.6	1230.8	1204.7
CCDC149	92.1	88.9	73.2	107.2	127.8	106.8
CCDC69	111.1	85.4	93.9	161.4	148.0	193.6
CCNG2	32.3	18.5	20.6	37.2	40.5	44.0
CDCA7L	25.6	37.0	27.2	39.5	54.3	56.5
CDRT1	197.6	169.9	233.7	259.6	258.7	295.1
CENPA	143.4	148.8	149.2	203.2	197.0	199.9
CERS6-AS1	66.5	65.1	81.7	109.5	123.5	64.9
CFB	171.9	235.0	246.8	269.8	280.0	355.9
CHKB	85.5	85.4	96.7	106.1	146.9	103.6
CHST11	35.1	44.0	49.7	73.4	73.5	57.6
CLDN2	171.0	147.0	137.0	214.4	211.9	219.8
CLDN3	67.4	73.9	77.9	102.7	113.9	102.6
CMTM3	79.8	102.1	104.2	140.0	131.0	106.8
CPN2	77.9	76.6	77.0	88.0	107.5	121.4
CPNE2	78.8	74.8	64.8	138.8	123.5	103.6
CRIP2	2.8	4.4	5.6	15.8	16.0	8.4
CROT	970.7	954.3	855.0	1238.2	1182.9	1243.4
CTNNBIP1	137.7	106.5	92.9	221.2	185.3	209.3
CTSF	206.1	170.8	187.7	281.0	255.5	311.9
CYP4A11	8.5	6.2	14.1	21.4	36.2	39.8
CYP4V2	239.3	245.6	241.2	434.5	391.8	393.5
CYSRT1	8.5	2.6	2.8	14.7	16.0	8.4
DAP	1278.4	1313.5	1352.4	1811.5	1739.7	1716.5
DCAF10	571.8	580.1	476.8	699.8	683.5	751.5
DHX32	280.2	305.5	305.0	418.7	436.5	375.7
DNAH1	125.4	114.4	131.4	160.3	176.7	180.0

DPP10	158.6	176.9	162.4	235.9	241.7	278.4
DPYD	51.3	51.9	60.1	93.7	85.2	106.8
DPYS	35.1	26.4	16.0	51.9	62.8	62.8
DSEL	74.1	78.3	74.1	126.4	112.9	127.7
ECHDC3	135.8	152.3	138.9	219.0	209.7	229.2
EDIL3	19.9	25.5	12.2	36.1	34.1	33.5
EHD2	16.1	22.9	17.8	39.5	43.7	22.0
ERBB2	423.6	453.4	456.1	576.8	616.5	562.0
ERO1B	11.4	7.9	5.6	24.8	36.2	38.7
ERV3-1	17.1	10.6	13.1	32.7	23.4	19.9
EVL	96.9	73.1	89.2	135.4	138.4	124.5
F12	87.4	105.6	108.9	169.3	183.1	185.3
F13B	40.8	37.0	29.1	60.9	55.4	67.0
FAM110A	52.2	43.1	42.2	67.7	59.6	69.1
FAM129A	20.9	7.0	10.3	38.4	43.7	29.3
FAM132B	1.9	0.9	3.8	10.2	11.7	4.2
FAM20A	75.0	70.4	70.4	155.8	115.0	131.9
FAM84B	26.6	15.0	16.9	29.3	41.5	51.3
FARP1	417.9	458.7	419.5	540.6	635.6	539.0
FBP1	99.7	94.2	92.9	150.1	159.7	154.9
FCGBP	153.9	146.1	195.2	205.4	220.4	225.0
FTCD	15.2	22.0	25.3	42.9	33.0	33.5
GPR176	23.7	49.3	40.4	51.9	62.8	57.6
GPRASP2	98.8	80.1	127.6	136.6	125.6	139.2
GREM1	83.6	86.3	78.8	124.2	89.4	122.5
GRIA3	138.7	125.9	175.5	212.2	189.5	230.3
GTF2IRD2	171.0	144.4	147.4	207.7	220.4	210.4
GTF2IRD2B	61.7	50.2	60.1	88.0	87.3	94.2
GUCY1A2	10.4	3.5	5.6	13.5	18.1	17.8
H6PD	681.0	669.9	711.4	972.9	1027.4	1065.5
HADH	299.2	304.6	304.1	421.0	391.8	383.1
HEG1	134.9	132.9	93.9	165.9	154.4	220.8
HERPUD1	474.9	484.2	432.7	653.5	632.4	581.9
HGSNAT	224.2	221.0	231.8	401.8	376.9	357.9
HMGCS2	1609.9	1505.4	1508.2	1997.8	2085.8	2337.1
HOGA1	24.7	48.4	30.0	55.3	58.6	83.7

HRG	76.9	81.9	100.4	125.3	128.8	194.7
HS3ST3B1	253.6	261.5	256.2	340.9	343.9	332.8
HSD17B8	74.1	85.4	90.1	100.5	101.1	145.5
HSPA5	8504.4	8900.2	8878.6	11810.5	11744.8	12540.6
HSPG2	429.3	444.6	504.0	608.4	688.9	584.0
HYOU1	2436.2	2403.3	2497.5	3241.6	3411.3	3259.2
IDUA	32.3	21.1	39.4	44.0	61.8	41.9
IGFBP1	109.2	101.2	126.7	194.1	182.1	174.8
IGFBP3	464.4	482.4	515.3	793.5	700.6	721.1
IGFBP5	90.2	94.2	82.6	205.4	189.5	219.8
IGSF1	38.9	46.7	34.7	62.1	70.3	75.4
IRS2	129.2	132.1	96.7	189.6	162.9	187.3
ITIH1	163.4	164.6	150.2	199.8	236.4	311.9
ITPR2	521.4	480.7	467.4	574.5	619.7	728.4
KCTD14	39.9	47.5	65.7	49.7	109.7	85.8
KIF20A	1022.9	1130.4	1089.7	1397.3	1389.4	1431.8
KLF11	221.3	247.4	220.6	326.2	287.5	308.8
KLF15	103.5	120.6	83.5	159.1	141.6	150.7
KNG1	155.8	141.7	160.5	216.7	234.2	277.4
KREMEN1	28.5	24.6	18.8	31.6	61.8	29.3
LAMP3	7.6	12.3	16.0	31.6	31.9	29.3
LARP6	51.3	59.0	46.9	91.4	80.9	63.8
LHPP	60.8	88.9	65.7	107.2	125.6	100.5
LOC285074	144.4	164.6	146.4	182.8	216.1	202.0
LOC400043	195.7	161.1	191.5	311.5	282.1	211.4
LOC653160	5.7	8.8	9.4	12.4	20.2	29.3
LPPR4	69.3	70.4	68.5	146.7	103.3	142.3
LRP3	597.4	671.7	628.8	963.9	972.1	933.6
LRRC48	5.7	4.4	9.4	9.0	12.8	24.1
LSR	246.0	257.9	264.7	358.9	337.5	314.0
LTBP2	34.2	44.0	40.4	58.7	62.8	59.7
LYZ	17.1	34.3	25.3	55.3	50.0	67.0
MAMLD1	46.5	36.1	40.4	108.4	128.8	123.5
MAP3K15	23.7	16.7	22.5	38.4	36.2	48.1
MATN2	322.9	324.0	367.0	568.9	597.3	542.2
MESP1	45.6	40.5	31.9	53.0	63.9	68.0

MEST	190.9	199.8	146.4	239.3	250.2	235.5
MEX3A	387.5	373.3	377.3	474.0	483.4	527.5
MFI2	260.2	219.2	278.7	393.9	342.8	355.9
MFSD6	144.4	118.0	126.7	191.9	210.8	177.9
MLXIPL	289.7	256.2	321.9	347.6	412.0	367.4
MSI1	3.8	10.6	3.8	16.9	20.2	15.7
MYCL	20.9	15.0	34.7	45.1	43.7	44.0
MYH3	69.3	63.4	65.7	89.2	95.8	97.3
MYO1D	593.6	623.3	535.9	758.5	760.2	884.4
MYO9A	122.5	123.2	104.2	158.0	157.6	168.5
NEURL2	84.5	79.2	91.0	121.9	111.8	110.9
NIPAL2	118.7	82.8	92.9	153.5	141.6	140.2
NMNAT2	3.8	0.9	6.6	13.5	12.8	11.5
NR1D1	97.8	110.0	97.6	142.2	168.2	126.6
NR3C2	51.3	51.9	47.9	74.5	71.3	71.2
NUCB2	256.4	238.6	229.0	302.5	330.1	308.8
OLFM2	77.9	89.8	83.5	125.3	110.7	101.5
ONECUT1	228.0	218.3	254.3	279.9	356.7	280.5
OR2A4	70.3	67.8	49.7	93.7	102.2	83.7
ORAI3	62.7	71.3	76.0	93.7	100.1	96.3
OTC	120.6	134.7	102.3	156.9	173.5	192.6
P2RY11	5.7	18.5	31.9	37.2	28.7	51.3
PALMD	74.1	69.5	76.0	114.0	101.1	114.1
PAPPA	1.9	5.3	0.9	9.0	11.7	8.4
PARD3B	102.6	120.6	90.1	172.7	133.1	164.3
PDK4	255.5	225.4	228.1	416.5	369.5	393.5
PGLS	255.5	236.8	312.5	431.2	370.5	354.8
PHF1	211.8	188.4	205.5	251.7	266.2	274.2
PHYH	444.5	439.3	398.9	601.6	609.0	534.8
PIF1	115.9	113.6	139.8	173.8	181.0	152.8
PIGZ	175.7	200.7	252.5	321.7	270.4	285.7
PKN1	651.6	603.9	586.6	843.1	834.7	802.8
PLG	27.5	25.5	28.2	47.4	71.3	76.4
PLXNA3	283.0	253.5	317.2	393.9	381.2	359.0
PPP1R9A	28.5	18.5	28.2	48.5	42.6	47.1
PRR36	2.8	0.9	1.9	4.5	12.8	5.2

RBPMS2	4.7	5.3	2.8	9.0	21.3	9.4
REEP6	327.7	339.8	394.2	518.1	549.4	534.8
REG4	0.0	1.8	1.9	9.0	5.3	7.3
RELN	87.4	97.7	107.0	152.4	177.8	205.1
REPS2	0.9	0.0	0.9	6.8	6.4	5.2
RFX6	28.5	37.9	26.3	60.9	68.1	54.4
RIN3	17.1	29.9	27.2	45.1	40.5	55.5
RTEL1-TNFRSF6B	38.9	53.7	39.4	70.0	113.9	54.4
RTKN2	64.6	75.7	57.3	98.2	85.2	92.1
SCARF2	6.6	2.6	8.4	12.4	13.8	16.7
SEMA6A	54.1	46.7	57.3	97.1	76.7	86.9
SEPT6	55.1	64.3	52.6	84.7	73.5	94.2
SERPINC1	320.1	374.1	387.6	445.8	467.4	536.9
SHC2	40.8	25.5	14.1	55.3	37.3	44.0
SHF	88.3	81.9	71.3	110.6	102.2	124.5
SIPA1L2	25.6	27.3	38.5	56.4	44.7	45.0
SLC13A5	12.3	8.8	8.4	19.2	24.5	18.8
SLC17A4	63.6	62.5	70.4	107.2	102.2	87.9
SLC35G2	6.6	7.9	4.7	16.9	9.6	19.9
SLC6A1	52.2	51.1	60.1	116.3	148.0	177.9
SLC6A15	1.9	2.6	3.8	5.6	11.7	11.5
SRD5A3	204.2	170.8	162.4	247.2	252.3	221.9
ST3GAL3	129.2	121.5	126.7	160.3	170.4	184.2
STOX1	53.2	58.1	41.3	81.3	101.1	82.7
SULT2A1	1.9	3.5	3.8	7.9	13.8	9.4
SYT15	32.3	27.3	36.6	63.2	59.6	63.8
TCFL5	228.9	255.3	217.7	295.7	313.0	319.2
TEX15	12.3	9.7	8.4	22.6	23.4	36.6
THBS3	57.0	57.2	68.5	74.5	85.2	94.2
TMEM132A	134.9	148.8	185.8	232.5	237.4	202.0
TMEM183A	124.4	185.8	152.0	272.0	177.8	297.2
TMEM79	109.2	116.2	104.2	143.3	151.2	136.1
TPRN	382.8	337.2	439.2	530.5	497.2	495.1
TTC7B	23.7	36.1	30.0	47.4	50.0	47.1
TTR	127.3	130.3	158.6	205.4	197.0	222.9
TTYH3	161.5	150.5	139.8	206.5	235.3	211.4

TYSND1	491.0	483.3	488.0	732.5	703.8	669.8
UBAP1L	0.9	3.5	3.8	7.9	10.6	9.4
UGT1A4	45.6	27.3	36.6	71.1	54.3	55.5
UGT2B15	130.1	106.5	116.4	199.8	168.2	192.6
ULK2	70.3	86.3	55.4	121.9	79.9	91.1
UNC119B	488.2	487.7	500.2	637.7	675.0	621.7
VASN	23.7	29.1	29.1	41.8	35.1	58.6
VCAM1	407.5	426.1	356.6	556.4	541.9	480.4
VIM-AS1	13.3	13.2	7.5	38179.8	37221.2	38960.5
WASF1	470.1	500.0	529.3	663.7	633.5	673.0
WWC3	117.8	105.6	111.7	258.5	239.6	248.0
XK	37.0	44.9	54.4	93.7	75.6	76.4
ZBTB8A	49.4	52.8	68.5	95.9	91.6	80.6
ZCCHC14	642.1	613.6	552.8	839.7	810.2	831.0
ZIC2	6.6	2.6	7.5	14.7	16.0	26.2
ZNF256	25.6	16.7	24.4	36.1	38.3	34.5
ZNF362	96.9	87.2	111.7	147.9	144.8	146.5
ZNF496	94.0	82.8	70.4	115.1	111.8	115.1
ZNF503	30.4	38.7	35.7	60.9	54.3	57.6
ZNF837	10.4	7.9	19.7	32.7	27.7	23.0

Supplementary Table 2. List of genes differentially expressed after *VIM-AS1* and *EPHA3* co-overexpression.

[Down-regulated genes]

Gene Name	CON1	CON2	CON3	VIM-AS1/ EPHA3 OE1	VIM-AS1/ EPHA3 OE2	VIM-AS1/ EPHA3 OE3
ABCB5	2.0	2.9	2.8	6.6	11.3	8.4
ACKR3	23.5	18.3	15.2	35.5	41.9	44.6
ADAM20	1.0	1.0	0.0	0.0	18303.2	18038.0
ADAM23	59.3	78.0	74.0	180.6	157.3	166.6
ADM2	74.7	92.5	52.2	111.9	114.3	129.6
ADSSL1	7.2	5.8	2.8	17.7	17.0	10.9
AFF3	269.0	282.3	267.7	346.9	376.8	346.7
AGR3	0.0	1.0	0.9	268.2	1.1	290.3
AHRR	9.2	18.3	10.4	27.7	22.6	23.6
AJUBA	1382.0	1286.2	1131.6	1732.1	1656.5	1707.4
ALOX5	1.0	1.0	0.9	221.6	1.1	213.7
ANKRD20A19P	1.0	1.0	0.9	1.1	503.5	488.9
ANKRD36BP1	93.1	95.4	96.8	114.1	125.6	142.2
ANO4	13.3	8.7	6.6	26.6	18.1	16.0
ANXA10	56.3	55.9	58.9	120.8	89.4	70.7
ARFGEF3	44.0	52.0	55.1	74.2	81.5	68.2
ARHGAP25	1.0	1.0	0.0	1.1	177.6	159.0
ASPHD2	2.0	1.9	1.9	10.0	4.5	9.3
ATF3	133.0	146.4	123.4	262.6	272.7	216.3
B3GALT4	4.1	2.9	3.8	16.6	13.6	16.8
BBS5	6.1	2.9	3.8	21.1	17.0	18.5
BCL11B	57.3	68.4	74.0	72.0	114.3	93.4
BDNF	0.0	1.0	0.9	123.0	122.2	0.0
BTN1A1	1.0	1.0	0.9	187.3	0.0	180.1
C11orf96	1.0	0.0	0.9	0.0	217.2	209.5
C1QTNF3	13.3	12.5	21.8	34.4	30.6	29.5
CACNA1G	52.2	55.9	52.2	114.1	89.4	93.4
CAPN9	134.0	126.2	127.2	297.0	208.2	227.2
CCDC144B	7.2	1.9	5.7	21.1	10.2	11.8
CEACAM6	115.6	96.3	86.4	205.0	196.9	203.6

CENPI	121.7	106.0	105.4	141.8	159.5	140.5
CEP68	172.9	143.6	156.6	209.4	207.1	209.5
CERKL	3.1	1.9	3.8	11.1	11.3	7.6
CGB5	1.0	1.0	0.9	1189.1	1146.2	1058.6
CHAC1	62.4	53.0	55.1	79.8	80.3	85.0
CHKB-AS1	3.1	1.9	0.9	16.6	12.4	9.3
CITED1	0.0	1.9	1.9	6.6	6.8	10.1
CNTN1	21.5	18.3	20.9	48.8	52.0	46.3
COLCA1	218.9	212.9	207.9	397.8	449.2	359.3
CPNE7	12.3	15.4	16.1	26.6	26.0	26.9
CRIP1	114.6	140.7	120.6	215.0	205.9	201.1
CT45A10	38.9	41.4	39.9	69.8	61.1	85.0
CTAG2	927.8	940.4	950.3	1326.5	1241.3	1260.5
CXCL2	28.6	41.4	23.7	66.5	71.3	52.2
CYP26B1	91.0	83.8	64.6	137.4	110.9	129.6
CYR61	477.7	444.2	370.2	660.5	638.2	621.9
DIO2	71.6	68.4	49.4	86.4	92.8	83.3
DLG3	21.5	24.1	19.0	32.1	38.5	44.6
DUSP5	226.1	202.3	219.3	330.2	316.8	323.1
EDN1	242.4	227.4	220.2	375.7	347.4	297.0
EFNB1	149.4	133.0	158.5	228.3	226.3	209.5
ELFN2	75.7	80.0	82.6	116.4	98.4	130.4
EMP1	166.7	175.4	122.5	206.1	204.8	198.6
ENO2	10.2	14.5	19.0	29.9	24.9	30.3
EPHA3	2.0	2.9	1.9	36.6	49.8	37.0
ETNPPL	120.7	126.2	147.1	179.5	189.0	168.3
FAT3	43.0	38.5	42.7	66.5	47.5	74.9
FBXL14	39.9	50.1	47.5	74.2	54.3	69.8
FOS	1091.5	1048.3	1280.6	1641.2	1676.9	1616.5
FOSB	128.9	113.7	132.9	299.2	340.6	262.5
FOSL2	91.0	104.1	124.4	164.0	155.0	148.9
FTX	1.0	4.8	5.7	18.8	15.8	14.3
G6PC	20.5	16.4	26.6	34.4	35.1	39.5
GADD45B	258.8	275.6	273.4	387.9	346.2	367.7
GLB1L3	6.1	10.6	12.3	14.4	29.4	27.8
GLDN	196.4	157.0	153.8	238.3	297.6	246.6

GOLGA2P10	239.4	239.9	244.0	300.3	302.1	356.8
GPR161	14.3	20.2	13.3	39.9	38.5	39.5
GPRC5B	184.1	185.0	123.4	275.9	235.4	281.1
GPSM1	18.4	15.4	11.4	41.0	37.3	31.1
GSTT2B	84.9	74.2	80.7	137.4	146.0	84.1
GTSF1	51.1	60.7	68.3	107.5	82.6	79.9
HARBI1	35.8	24.1	32.3	61.0	39.6	55.5
HES1	841.9	828.6	894.2	1268.9	1306.9	1109.9
HSPB3	1.0	1.0	0.0	0.0	228.6	220.5
IGFBP1	90.0	82.9	78.8	117.5	118.8	129.6
IGFBP2	81.8	93.5	84.5	187.3	155.0	175.0
IGFBP3	468.5	409.5	423.4	674.9	762.6	719.5
IL11	1.0	1.0	0.0	30660.2	33408.7	0.8
IRF8	37.8	46.2	44.6	66.5	63.4	64.8
JDP2	50.1	49.1	50.3	83.1	64.5	73.2
KCNK5	41.9	44.3	41.8	73.1	67.9	62.3
KLF2	254.7	274.6	263.9	465.4	400.6	462.0
KLF6	861.3	801.6	907.5	1204.6	1176.8	1149.5
KRT19	796.9	827.6	630.3	991.8	944.8	1034.2
KRT4	68.5	59.7	40.8	145.2	142.6	159.9
KRT7	2439.8	1959.7	1361.3	3439.8	2696.4	2984.7
KRT80	336.6	276.5	194.6	566.3	510.3	525.1
KRT83	24.6	24.1	14.2	38.8	27.2	46.3
LBH	47.1	60.7	38.0	95.3	109.8	111.1
LGALS8-AS1	1.0	1.0	0.9	102.0	0.0	99.3
LINC00467	27.6	38.5	40.8	61.0	44.1	58.1
LINC00648	46.0	53.0	38.9	79.8	61.1	67.3
LINC00668	4.1	4.8	1.9	13.3	11.3	8.4
LINC01029	60.4	58.8	75.9	79.8	109.8	91.7
LINC01420	0.0	1.9	3.8	10.0	10.2	6.7
LOC100133331	493.1	424.9	473.7	585.1	750.2	606.7
LOC100506606	10.2	11.6	11.4	25.5	31.7	20.2
LOC101927018	1.0	1.0	0.0	654.9	624.6	575.6
LOC101928847	2.0	1.9	5.7	18.8	12.4	14.3
LOC102723927	1.0	1.0	0.0	275.9	275.0	249.1
LOC284454	52.2	60.7	47.5	73.1	103.0	77.4

LPPR3	3.1	1.0	3.8	7.8	5.7	16.0
LUM	242.4	230.3	248.7	430.0	440.2	427.5
MAFF	37.8	24.1	43.7	80.9	56.6	69.0
MAGEA11	17.4	27.9	31.3	53.2	65.6	65.6
MAGEA2	583.1	652.3	797.4	1056.1	772.8	1111.6
MARVELD2	110.5	87.7	114.9	144.1	139.2	152.3
MFSD6	84.9	95.4	77.8	110.8	114.3	117.0
MLPH	195.4	195.6	240.2	373.5	324.7	326.5
MMP28	19.4	18.3	19.9	43.2	24.9	40.4
MTCL1	139.1	126.2	115.8	172.9	149.4	176.7
MUC1	40.9	38.5	30.4	59.8	47.5	63.1
MYEOV	152.4	135.9	109.2	186.2	157.3	191.0
NBPF14	64.4	43.4	55.1	99.7	67.9	95.1
NCAM1	426.6	426.8	418.6	585.1	618.9	584.0
NCR3LG1	19.4	14.5	13.3	33.2	23.8	32.0
NEFH	175.9	177.3	168.0	335.8	322.5	340.8
NEMP2	109.5	106.0	90.2	133.0	186.7	166.6
NFKBID	41.9	32.8	46.5	61.0	69.0	53.0
NIPAL1	3.1	6.7	10.4	29.9	17.0	18.5
NR1D1	83.9	102.1	74.0	110.8	126.7	117.0
NR4A1	133.0	115.6	127.2	270.4	239.9	252.4
NR4A3	3.1	1.9	0.9	10.0	9.1	9.3
NRP2	60.4	74.2	60.8	119.7	95.0	84.1
NTN1	90.0	83.8	72.1	117.5	96.2	124.5
NUDT4P2	392.8	342.0	300.0	1231.2	1042.1	948.4
PADI2	0.0	1.0	0.9	0.0	122.2	117.8
PALM	157.5	168.6	184.2	241.6	255.7	247.4
PAPPA	2.0	2.9	1.9	0.0	14.7	14.3
PART1	100.3	93.5	75.0	108.6	131.3	119.5
PBDC1	177.0	234.1	216.4	295.9	279.5	277.7
PJA1	99.2	87.7	80.7	148.5	99.6	128.7
PLCH1	63.4	82.9	53.2	90.9	122.2	90.9
PLCXD2	13.3	10.6	15.2	26.6	23.8	29.5
PMS2P7	28.6	26.0	33.2	41.0	50.9	44.6
PPP1R15A	599.5	614.7	615.1	889.9	843.0	843.2
PPP1R1A	1.0	4.8	0.9	12.2	14.7	15.1

PRAME	10.2	3.9	9.5	24.4	22.6	16.8
PRKAR2A	270.1	283.3	294.3	377.9	319.1	418.2
PRKCB	4.1	2.9	7.6	16.6	19.2	16.0
PRR4	2.0	1.9	1.9	11.1	3.4	11.8
PTPRR	28.6	30.8	20.9	64.3	54.3	51.3
QPCT	47.1	40.5	44.6	52.1	72.4	68.2
RAB43	188.2	187.9	173.7	403.4	470.7	302.1
RASD1	68.5	59.7	68.3	113.0	95.0	109.4
RASEF	2.0	1.0	4.7	16.6	12.4	6.7
RASGEF1B	19.4	25.1	25.6	54.3	40.7	35.3
RELN	12.3	18.3	24.7	61.0	48.7	54.7
RFPL1S	8.2	8.7	7.6	21.1	21.5	21.9
RGCC	0.0	3.9	2.8	7.8	7.9	12.6
RGPD6	0.0	1.0	1.9	601.7	551.0	0.0
RGS17	14.3	10.6	12.3	22.2	29.4	26.1
RND1	75.7	82.9	74.0	104.2	96.2	119.5
RPL22L1	425.6	467.3	399.7	527.5	617.8	555.4
RRN3P3	31.7	28.9	27.5	39.9	39.6	59.7
RTN1	1.0	1.0	0.9	968.6	1065.9	0.8
S100A2	45.0	37.6	40.8	61.0	55.4	82.5
SAGE1	179.0	161.9	162.3	229.4	235.4	231.4
SBK3	1.0	1.0	0.9	506.4	1.1	520.0
SEMA4A	1.0	0.0	0.9	146.3	146.0	0.0
SGK223	83.9	79.0	95.9	117.5	117.7	138.8
SIK1	784.6	780.4	622.7	1101.5	1078.3	948.4
SLC1A4	151.4	134.9	127.2	220.5	174.3	172.5
SLC46A3	6.1	1.9	14.2	11.1	24.9	21.0
SMYD3	237.3	235.1	235.4	329.1	332.7	351.7
SOGA1	225.1	239.9	268.7	307.0	330.4	346.7
SPAG5-AS1	2.0	1.9	2.8	7.8	6.8	10.9
SSX2	175.9	154.2	238.3	281.5	329.3	298.7
STC2	52.2	29.9	31.3	107.5	107.5	82.5
STKLD1	1.0	3.9	2.8	10.0	6.8	12.6
SYTL2	45.0	62.6	50.3	83.1	86.0	108.6
TAF7L	0.0	1.0	0.9	22624.7	22442.2	0.8
TFF2	4.1	3.9	1.9	10.0	9.1	13.5

TGFB2	171.9	190.8	157.6	291.5	196.9	226.4
THBD	18.4	23.1	15.2	34.4	41.9	33.7
THBS1	2602.4	2473.2	2188.1	3504.1	3351.5	3145.5
TNFRSF10A	39.9	55.9	57.0	84.2	76.9	87.5
TNFRSF11B	225.1	189.8	232.6	334.7	279.5	324.0
TPPP	28.6	16.4	22.8	35.5	35.1	62.3
TREML3P	3.1	1.9	1.9	11.1	9.1	19.4
TSC22D3	12.3	4.8	8.5	18.8	33.9	26.1
TTC22	116.6	117.5	153.8	187.3	132.4	192.7
TUBB6	98.2	73.2	75.9	164.0	161.8	152.3
VCAM1	185.2	179.2	154.7	236.0	204.8	237.3
ZFP92	1.0	3.9	0.9	14.4	0.0	13.5
ZNF256	26.6	28.9	25.6	39.9	41.9	48.0
ZNF496	44.0	42.4	43.7	70.9	64.5	68.2
ZNF551	122.8	119.5	109.2	137.4	149.4	171.7
ZNF813	27.6	32.8	29.4	43.2	60.0	48.8
ZSWIM5	35.8	26.0	24.7	42.1	57.7	38.7

[Up-regulated genes]

**Gene Name**	**CON1**	**CON2**	**CON3**	**VIM-AS1/ EPHA3 OE1**	**VIM-AS1/ EPHA3 OE2**	**VIM-AS1/ EPHA3 OE3**
AASS	55.2	64.6	69.3	31.0	44.1	32.0
ABAT	83.9	106.0	132.9	67.6	73.5	81.6
ACOT12	246.5	231.2	311.4	205.0	195.7	198.6
ACTL8	191.3	208.1	164.2	147.4	130.1	135.5
ADH1A	116.6	103.1	132.9	83.1	66.8	79.1
ADH1B	148.3	142.6	157.6	85.3	121.1	102.7
ADH4	136.1	114.7	141.4	93.1	113.2	79.1
AHSG	9916.6	10606.9	12418.8	8477.6	7508.6	8569.7
ALDOB	420.4	412.4	450.0	315.8	320.2	344.2
ANTXR2	66.5	83.8	85.4	56.5	60.0	51.3
APOA1	1615.3	1805.6	1874.9	1374.1	1371.4	1307.7
APOM	182.1	189.8	202.2	128.5	140.3	154.0
ARVCF	41.9	54.9	59.8	33.2	33.9	23.6
BCAM	81.8	80.9	91.1	65.4	36.2	59.7

CALB2	27.6	33.7	20.9	14.4	10.2	16.0
CCDC149	95.1	112.7	94.0	70.9	75.8	61.4
CD44	164.7	141.6	113.0	107.5	100.7	101.0
CFHR2	156.5	163.8	185.1	113.0	144.8	130.4
CPS1	10295.1	10721.6	13634.8	8804.5	9079.2	8478.8
CTSO	19.4	12.5	10.4	5.5	4.5	5.9
DPYD	91.0	82.9	103.5	59.8	56.6	59.7
ERN1	59.3	77.1	80.7	47.7	54.3	47.1
FAM45A	342.7	441.3	361.7	254.9	318.0	248.2
FFAR4	14.3	15.4	10.4	5.5	4.5	5.0
FMO1	5.1	6.7	8.5	0.0	1.1	0.8
FSCN1	135.0	123.3	93.0	80.9	75.8	77.4
FZD1	93.1	113.7	80.7	62.1	83.7	62.3
GUSBP2	11.3	13.5	7.6	3.3	5.7	2.5
IFIT1	54.2	58.8	57.0	37.7	35.1	42.1
IHH	123.8	135.9	147.1	117.5	93.9	91.7
IQCH-AS1	46.0	53.0	56.0	36.6	27.2	35.3
ISY1-RAB43	509.4	543.4	607.6	331.3	222.9	452.7
KDM7A	117.6	131.0	172.8	92.0	135.8	79.9
KLF15	181.1	161.9	171.8	107.5	110.9	127.9
KLHL28	156.5	141.6	188.0	117.5	123.3	122.9
KNG1	386.7	414.3	483.2	285.9	312.3	302.9
KRT20	37.8	45.3	50.3	12.2	10.2	11.8
LINC00638	5.1	6.7	5.7	0.0	1.1	0.8
LOC727751	74.7	105.0	109.2	66.5	47.5	70.7
LRRC37A4P	93.1	101.2	97.8	66.5	79.2	71.5
MAPK13	156.5	167.6	173.7	118.6	113.2	125.4
MGAT4A	369.3	313.1	317.1	261.5	272.7	220.5
MYB	73.7	84.8	84.5	51.0	49.8	65.6
NEB	1344.2	1384.5	1652.7	1087.1	1133.8	1103.2
PCK1	8.2	4.8	10.4	1.1	1.1	0.0
PDK4	200.5	174.4	188.0	137.4	135.8	152.3
PKLR	183.1	217.7	233.5	151.8	157.3	131.3
RBMY1F	33.8	11.6	23.7	5.5	11.3	10.1
RILPL2	114.6	110.8	114.9	78.7	79.2	88.4
SERPINC1	363.2	380.6	439.5	304.8	285.1	301.3

SERPINF1	181.1	163.8	184.2	127.4	117.7	121.2
SLC25A27	84.9	75.2	83.5	45.4	63.4	57.2
SLC3A1	264.9	225.5	297.1	191.7	194.6	217.1
SLC6A1	27.6	40.5	53.2	24.4	18.1	20.2
STARD4-AS1	101.3	85.7	103.5	74.2	74.7	54.7
TMEM170B	65.5	53.0	70.2	47.7	31.7	40.4
TNRC6C	73.7	69.4	54.1	42.1	41.9	41.2
TP63	54.2	36.6	30.4	10.0	12.4	10.1
VGLL3	43.0	62.6	61.7	21.1	28.3	25.2
